# Exploring Potential of Pearl Millet Germplasm Association Panel for Association Mapping of Drought Tolerance Traits

**DOI:** 10.1371/journal.pone.0122165

**Published:** 2015-05-13

**Authors:** Deepmala Sehgal, Leif Skot, Richa Singh, Rakesh Kumar Srivastava, Sankar Prasad Das, Jyoti Taunk, Parbodh C. Sharma, Ram Pal, Bhasker Raj, Charles T. Hash, Rattan S. Yadav

**Affiliations:** 1 Institute of Biological, Environmental and Biological Sciences (IBERS), Aberystwyth University, Gogerddan, Aberystwyth, Ceredigion, United Kingdom; 2 Chaudhary Charan Singh Haryana Agricultural University (CCSHAU), Department of Molecular Biology and Biotechnology, Hisar, Haryana, India; 3 International Crops Research Institute for the Semi-Arid Tropics (ICRISAT), Patancheru, Andhra Pradesh, India; 4 ICAR Research Complex for NEH Region, Tripura Centre, Lembucherra, India; 5 Central Soil Salinity Research Institute (CSSRI), Karnal, India; 6 National Research Centre for Orchids, Darjeeling Campus, Darjeeling, India; 7 ICRISAT Sahelian Center, BP 12404, Niamey, Niger; National Institute of Plant Genome Research (NIPGR), INDIA

## Abstract

A pearl millet inbred germplasm association panel (PMiGAP) comprising 250 inbred lines, representative of cultivated germplasm from Africa and Asia, elite improved open-pollinated cultivars, hybrid parental inbreds and inbred mapping population parents, was recently established. This study presents the first report of genetic diversity in PMiGAP and its exploitation for association mapping of drought tolerance traits. For diversity and genetic structure analysis, PMiGAP was genotyped with 37 SSR and CISP markers representing all seven linkage groups. For association analysis, it was phenotyped for yield and yield components and morpho-physiological traits under both well-watered and drought conditions, and genotyped with SNPs and InDels from seventeen genes underlying a major validated drought tolerance (DT) QTL. The average gene diversity in PMiGAP was 0.54. The STRUCTURE analysis revealed six subpopulations within PMiGAP. Significant associations were obtained for 22 SNPs and 3 InDels from 13 genes under different treatments. Seven SNPs associations from 5 genes were common under irrigated and one of the drought stress treatments. Most significantly, an important SNP in putative acetyl CoA carboxylase gene showed constitutive association with grain yield, grain harvest index and panicle yield under all treatments. An InDel in putative chlorophyll a/b binding protein gene was significantly associated with both stay-green and grain yield traits under drought stress. This can be used as a functional marker for selecting high yielding genotypes with ‘stay green’ phenotype under drought stress. The present study identified useful marker-trait associations of important agronomics traits under irrigated and drought stress conditions with genes underlying a major validated DT-QTL in pearl millet. Results suggest that PMiGAP is a useful panel for association mapping. Expression patterns of genes also shed light on some physiological mechanisms underlying pearl millet drought tolerance.

## Introduction

Pearl millet [*Pennisetum glaucum* (L.) R. Br.] is the sixth most important global cereal crop which is grown by subsistence farmers in the semi-arid regions of sub-Saharan Africa and the Indian subcontinent [1]. It is the main source of food for 500 million of the poorest people living predominantly in parts of Asia and Africa. It is the hardiest cereal crop that can be grown in a vast range of harsh environmental conditions, for instance, environments with high mean temperatures and frequent droughts and/or with poor soil fertility. Pearl millet grain has relatively high nutritional value compared to wheat, rice and maize in terms of both protein content and amino acid composition [2–4]. It also has superior levels of grain Fe and Zn [5, 6]. Furthermore, pearl millet also has relatively high energy density as compared to maize, wheat or sorghum [7].

A tremendous phenotypic variability exists in cultivated pearl millet for many agronomic traits such as flowering time, panicle length, grain and stover characteristics, and for tolerance to many biotic and abiotic stresses [8, 9]. Despite this, narrow gene pools are used for generating pearl millet varieties and hybrids and use of wild pearl millets and landrace germplasm, except as donors of specific traits such as apomixis [10] or resistance to pests and diseases [11], is very limited. Further, most of the allele mining for agronomically important traits including biotic and abiotic stress resistance has been achieved so far using bi-parental mapping populations [12–17]. For example, based on mapping in conventional bi-parental mapping populations, many quantitative trait loci (QTLs) for drought tolerance [14–16] downy mildew resistance [13] and yield and stover quality traits [18] have been identified over the past twenty years.

While conventional linkage mapping has identified a number of important quantitative traits in pearl millet, it has been severely limited by the resolution provided by two (or a few) parent-derived mapping populations. Due to small (79–125) population sizes and the early generations used, resolution has been in the range of 10-30cM in these studies. Therefore, there is need to explore and venture into improved alternatives for allele mining for improving pearl millet open-pollinated varieties and hybrids. Association mapping, also known as linkage disequilibrium (LD) mapping, offers an alternative means of allele mining, which utilizes ancestral recombination events in germplasm collections or natural populations to make marker-phenotype associations [19]. This approach has four major advantages over conventional QTL mapping. Firstly, a much larger and diverse genepool having thousands of recombination events is surveyed. Secondly, it saves time and labour that goes in designing mapping populations and enables the mapping of many traits in a single panel. Thirdly, the high mapping resolution achieved in association mapping results in small confidence intervals of the detected loci and sometimes even resolves the candidate quantitative trait gene (QTG). Finally, it has the potential to identify the causal polymorphism within a gene and/or quantitative trait nucleotide in a QTG linked with the two alternative phenotypes. Association mapping has been successfully used in many crops such as maize, barley, sorghum, rice, and common wheat to detect important markers or genes [20–26].

Recently, a pearl millet germplasm association panel (PMiGAP) comprising 250 inbred lines has been assembled from a large set of 1000 diverse breeding lines and accessions of landraces, elite cultivars and mapping population parents, collected from wide geographical range in Africa and Asia [27]. It is anticipated that PMiGAP will provide the pearl millet community with a high-resolution platform for fine mapping of QTLs and (or) for allele mining of favourable genes of agronomic importance. To test whether PMiGAP is a good panel for association analysis, candidate gene-based association mapping was performed using 17 genes underlying a major QTL for drought tolerance on linkage group 2 [28]. For diversity and genetic structure analysis, PMiGAP was genotyped with 37 SSR and CISP markers representing all seven linkage groups. For association analysis, it was phenotyped for yield and yield components and morpho-physiological traits under both well-watered and drought conditions, and genotyped with SNPs and InDel markers designed from seventeen genes in the present and our previous study [28].

## Materials and Methods

The permission to carry out field experiments was obtained from International Crops Research Institute for the Semi-Arid Tropics (ICRISAT), Patancheru, Hyderabad, India and phenotyping was carried out at fields of ICRISAT, Patancheru, Hyderabad, India.

### Plant material and DNA extraction

Two hundred and fifty inbred lines of PMiGAP, representing 23 countries across three continents (S1 Table), were used for association analysis. The PMiGAP has been developed from a pearl millet core collection [8, 29], landraces, cultivars and breeding lines representing entire cultivated global diversity of pearl millet. The selfing programme to develop inbred lines from these open-pollinated entries began in 2007/08 at ICRISAT using single row plots of 4-m length of each source population under standard agronomic practices to raise a healthy crop. One selfed panicle was harvested from each of three representative plants of each of the 250 accessions. The S1 seeds were harvested on a single-plant basis, and head-to-row progenies from each accession were grown in triplets of single-row plots of 4-m length during early post-rainy season in 2008. Three selfed panicles were harvested from one of the three representative S1 progenies to produce S2 seeds, with selection for slightly reduced vigour (to accelerate the rate of progress towards homozygosity), adequate selfed seed set, and typical plant architecture. Following the same methodology of selfing and selection, the generation was advanced to S3 (summer, 2009), S4 (late rainy season, 2009), and S5 (summer, 2010). During summer 2010, 250 PMiGAP-entries were testcrossed to one tester, ICMA 843–22, using bulk pollen from individual entries. Seeds of selfed inbred and test crosses were harvested and threshed. During 2010 late rainy season sowing, all 250 lines were sown as single-row plots of 4-m row length. Leaf tissue sampling at late seedling stage from five representative plants of each of the 250 entries was collected and bulked for DNA extraction and stored at -80°C. DNA was extracted from frozen leaf using DNeasy plant DNA kit (Qiagen, Hilden, Germany) and was quantified using a NanoDrop 1000 spectrophotometer.

### Phenotyping of PMiGAP under drought stress

The 250 PMiGAP entries were assigned to four precocity groups (61, 63, 63 and 63 entries in early, medium early, medium and late maturity groups, respectively) so that flowering time would not compound the results of drought tolerance. They were phenotyped as test cross hybrid trials during the summer seasons (January to May) of 2011 and 2012 in the experimental farm of the International Crops Research Institute for the Semi-Arid Tropics (ICRISAT), located in Patancheru, Telengana, India (altitude 545 m above mean sea level, latitude. 17.53° N and longitude 78.27° E). Both seasons experiments were laid out in alpha-lattice designs with two replications in 3 test environments. Individual plots were 4.0 m long; net (harvested) plot area was two rows by 3.0 by 0.6 m. Four checks; 843-22A x ICMR 01004, 843-22A x ICMR 01029, 863B x ICMR 01004 and 841A x D 23 were repeated across trials and used in calculating adjusted means of the traits. The 3 test environments consisted of two terminal drought stress treatments, an early-onset and a late-onset of terminal stress, plus a common, fully-irrigated non-stress treatment. Drought stress in the more severe early-onset treatment was initiated at 50% flowering, by withholding irrigation from about 1 week before flowering. Drought stress in the late-onset treatment was initiated during early grain filling by withholding irrigation from approximately 50% flowering. These entries were evaluated for 16 morphological, morpho-physiological and agronomical traits. The investigated traits were grain yield (GY), panicle yield (PY), panicle harvest index (PHI), time to 75% flowering (FT), plant height (PH), panicle length (PL), panicle diameter (PD), panicle number (PN), number of tillers per plant (TPP), biomass yield (BY), grain harvest index (GHI), thousand grain weight (TGW), grain number per panicle (GNPP), grain number per m^2^ (GNPM), stay green (SG) and leaf rolling (LR). These traits were measured as described in [14, 15]. Briefly, PH, PL, and PD were measured from main stems of five representative plants of each entry in a plot. At harvest, data were recorded from the harvested area on plant numbers (PC), head numbers (HC) and fresh stover yield (FSY). Effective tiller number (ET) was calculated as the ratio HC/PC. PY, GY and TGW were recorded after oven drying for approximately 24h. Stover dry matter yield (SDMY) was estimated from plot FSY using the fresh and dry weights of a chopped subsample of stover from each plot. BY was calculated as PY + SDMY on a plot basis. Panicle grain number (PGN) was derived from these primary data as (GY/HC)/ (TGM/1000). Grain harvest index represents the ratio between grain yield and biomass yield at harvest, and panicle harvest index the ratio between grain weight and panicle weight. Flowering time was recorded as days from seedling emergence to stigma emergence in 75% of the main shoots in a plot. Leaf rolling was measured after the day first symptom became visible on PMiGAP genotypes (normally after 10 days of the last irrigation) and continued until 80% of the leaves had rolled in the water stress treatment using a scale of 1 (20% leaf rolled) to 5 (80% and above rolled). Similarly, stay green was measured from the day leaf drying was visible using the scale of 1 (20% leaf staying green) to 5 (80% leaf staying green).

### Phenotypic data analyses

All phenotypic analysis was conducted using GenStat ver. 14^th^ Edition. The minimum, maximum and mean values of each trait in each environment were calculated using summary statistics option in GenStat. Normality of the data for each trait was checked by drawing normal plots. For each environment, the restricted maximum likelihood (REML) analysis was performed with replications as a fixed effect and entries as random effects. For combined environment analysis, REML model was used with environments and replications*environments as fixed effect and entries and entries*environments as random effects. The REML model produced best linear unbiased predictors (BLUPs) and variance components for all traits. The variance components were used for calculating broad sense heritability (h^2^) for traits in each environment and over combined environments. For each environment h^2^ was calculated as: h^2^ = V_g_/ (V_g_ + V_err_/r) where V_g_ is genotypic variance and V_err_ is the error variance and r = number of replications for a single environment. For combined environments, h^2^ was calculated as V_g_ / ((V_g_ + (V_GxE_ /n) + (V_err_ /nr)) where V_g_ is genotypic variance, V_GxE_ is genotype x environment interaction variance, V_err_ is the error variance, n = number of environments and r = number of replications. The phenotypic correlations among traits were obtained using Minitab 15 (http://www.minitab.com/en-IT/products/minitab/).

### Microsatellite genotyping

Genotyping of PMiGAP entries with genome wide SSR markers was done using M13 tailed (5’CACGACGTTGTAAAACGAC3’) forward primers as described previously [28]. The PCR products were resolved on an ABI 3730 DNA Sequencer (Applied Biosystems, CA, USA). The GeneMapper program, version 3.7 (Applied Biosystems, Foster City, CA, USA), was used for reading and scoring alleles.

### Genotyping with gene-based conserved intron spanning primers (CISP) and single nucleotide polymorphism (SNP) markers

Genotyping of PMiGAP entries with InDel markers was done as described by Sehgal *et al*. [28]. For SNP genotyping, the seventeen genes underlying major DT-QTL [28] were amplified and sequenced in 48 randomly selected entries using the protocols described in Sehgal *et al*. [28]. Sequences obtained from 48 genotypes were aligned using MACAW 2.05 software and the putative SNPs were also verified on the sequence chromatograms as described in Sehgal *et al*. [28]. All the SNPs obtained in individual genes were used for genotyping 250 PMiGAP entries using the KASPar genotyping system (Kbiosciences, UK).

### Model-based population structure analysis

The Bayesian model-based population structure analysis implemented in STRUCTURE v2.2 [30] was used to analyse the population genetic structure in PMiGAP. K (genetic groups) values from 1 to 15 were tested by applying the ‘no admixture’ and the ‘correlated allele frequency’ models [31]. Three independent runs were achieved for each K and replication number was set to 50,000 for the burn-in and the Markov chain Monte Carlo (MCMC) periods. Once all the runs were finished, a zip archive containing all of the results-f files was created and used as an input in Structure Harvester program (http://taylor0.biology.ucla.edu/structureHarvester/index.php) to estimate ∆*K* [32]. It is an ad hoc measure which identifies the number of subpopulations by estimating the rate of change in the log probability of data between successive K values.

### Genetic diversity assessment and cluster analysis

The assessment of genetic diversity was conducted using the software POPGENE version 1.32 [33]. The following genetic diversity parameters were used: average number of alleles per locus, effective number of alleles, observed heterozygosity, expected heterozygosity, Shannon’s information index and Nei’s genetic diversity index. Genetic distances between subpopulations were calculated with Nei’s parameter using the DARWin 5 programme [34]. Principal coordinate analysis (PCA) was also performed using DARWin 5.

### Linkage disequilibrium (LD) analysis

Linkage disequilibrium was estimated with the software program TASSEL 3.0 (http://www.maizegenetics.net). LD significance was determined with 100,000 permutations for each locus. The squared correlation coefficients (*r* ^*2*^) between loci were used for quantifying LD. LD was calculated for each candidate gene separately.

### Kinship matrix and marker-trait associations

A pairwise kinship matrix was estimated using the program SPAGeDi [35]. Negative values between pairs of individuals were set to 0 in the resulting matrix. Marker-trait associations were determined using TASSEL version 3.0 (http://www.maizegenetics.net), employing both the general linear model (based on the Q- or PCA-matrix) and the mixed linear model (based on Q- or PCA-matrix and the kinship matrix).

The significance of marker trait associations (MTAs) was initially based on FDR-adjusted *P* values [36] with cut off set at 0.05. However, FDR-adjusted *P-*values were found to be highly stringent. Hence considering the potential risk of type II error, we used another criterion as described in Pasam *et al*. [37]. Based on this approach, the *P*-values obtained within the bottom 0.1 percentile of the distribution are significant. A threshold *P*-values of 0.05 corresponded to the bottom 0.1 percentile in the present study, which was then used to declare significant MTAs.

### Semi-quantitative RT-PCR of candidate genes

Seeds of three contrasting genotypes, H 77/833-2 (drought susceptible), PRLT 2/89-33 (drought tolerant) and ICMR 01029 (near isogenic line introgressed with drought tolerance QTL on LG2 from drought tolerant parent PRLT 2/89-33) were sown in 8 inch pots filled with compost in a controlled environment tuned with the following conditions: 10 h light/14 h dark, temperature 20°C (night)/28°C (day) and relative humidity of 60% (day)/80% (night), suitable for pearl millet growth. Three replicates per genotype were sown in the pots and plants were grown till maturity. Water stress was initiated by withholding water supply on the 45^th^ day after sowing (a week after panicle emergence) and continued for 7 days. Leaves were harvested in liquid nitrogen from the plants in both control and stress treatments, and stored at -80°C. Total RNA was isolated from the frozen leaves of the drought-stressed and control materials using RNeasy Plant Mini Kit (Qiagen, UK) with manufacturer’s protocol. RNA was treated with RNase free DNase (Qiagen, UK) to remove genomic DNA contamination followed by inactivation of DNase with RNeasy Min Elute cleanup kit (Qiagen, UK) according to the manufacturer’s instructions. The quantification of the purified RNA was done at 260 nm in a spectrophotometer. The quality of the RNA was checked by both gel analysis, and 260/280 and 260/230 nm spectrophotometric ratios. The RNA was stored at -80°C before use. One micro gram of total RNA was used to synthesize cDNA using iScript^TM^ cDNA synthesis kit (Biorad, UK) as per manufacturer’s protocol. Real-time PCR was performed using the SYBR Green PCR master mix kit (Applied Biosystems, Foster City, CA, USA) according to the manufacturers’ recommendations. The following PCR program was used; 10 min at 95°C, 40 cycles at 95°C for 15 s, 54°C for 1 min and 72°C for 40 s, with a final cycle at 72°C for 7 min. Relative mRNA accumulation of each candidate sequence was compared to the control by using the standard comparative C_t_ (2^-∆∆Ct^) method. Average C_t_ value was calculated for 3 replicates for the housekeeping actin gene and for each candidate sequence in controlled and stressed cDNA samples. ∆C_t_ was then determined by taking a difference of the two values.

## Results

### Genetic diversity in pearl millet germplasm association panel (PMiGAP)

A total of 37 SSR and InDel markers, covering all the seven linkage groups, were used to evaluate the genetic diversity in PMiGAP. A total of 259 alleles, ranging from 2 (*Xibmsp09/AP*, *Xibmsp07/AP*, *Xibmcp11/AP* and *Xibmsp43/AP*) to 16 (*Xipes0236*), were detected across 250 pearl millet accessions which represent an average allele number (A) of 7 per locus (Table 1). When alleles with a frequency lower than 0.05 were excluded, A reduced to 117 alleles (3.16 alleles per locus). The average effective number of alleles (A_e_) for 37 markers was 2.71. The difference between A and A_e_ indicates that a large proportion of the alleles (54.8%) had frequencies lower than 5%, with 40.1% of them (57 alleles) present in a single genotype. The Shannon’s information index ranged from 0.20 (*Xicmp* 3056) to 2.21 (*Xpsmp*2203), with a mean of 1.06 for all loci. The Nei’s gene diversity ranged from 0.07 (*Xibmsp31/AP*) to 0.87 (Xpsmp2203), with a mean of 0.54 for all loci.

**Table 1:** Observed (A) and effective number of alleles (A_e_), Shannon’s information index (I) and Nei’s gene diversity (H) for 37 genome wide markers.

Marker	A	A_e_	I	H
Xibmsp09/AP	2	1.66	0.59	0.40
Xibmsp07/AP	2	1.86	0.65	0.46
Xibmcp03	5	1.75	0.69	0.42
Xicmp3017	6	2.81	1.18	0.64
Xicmp3086	5	1.99	0.94	0.49
Xipes0004	8	4.36	1.63	0.77
Xipes0017	15	2.74	1.35	0.63
Xipes0093	12	3.30	1.47	0.69
Xipes0109	10	4.10	1.60	0.75
Xipes0129	9	2.11	1.09	0.52
Xipes0180	13	3.28	1.39	0.69
Xipes0213	5	2.70	1.11	0.63
Xpsmp2085	15	3.35	1.53	0.70
Xpsmp2214	7	3.14	1.24	0.68
Xpsmp2227	8	1.75	0.86	0.43
Xipes0174	6	1.46	0.57	0.31
Xipes0206	11	3.67	1.49	0.72
Xpsmp2206	6	3.61	1.42	0.72
Xpsmp2059	7	2.20	1.04	0.54
Xipes0218	4	1.18	0.36	0.15
Xibmsp31/AP	3	1.73	0.67	0.42
Xipes0152	7	3.12	1.38	0.68
Xctm03	4	2.20	0.98	0.54
Xicmp3056	3	1.09	0.20	0.08
Xipes0117	3	2.02	0.72	0.50
Xibmcp11/AP	2	1.94	0.67	0.48
Xibmsp43/AP	2	1.78	0.63	0.43
Xipes0157	8	3.19	1.35	0.68
Xibmcp09	4	1.80	0.66	0.44
Xibmsp31/AP	3	1.08	0.19	0.07
Xibmcp08	4	1.63	0.61	0.38
Xibmcp11	2	1.80	0.63	0.44
Xicmp3002	8	2.11	1.00	0.52
Xipes0236	16	6.66	2.10	0.85
Xipes0076	10	4.47	1.72	0.77
Xpsmp2203	13	7.98	2.21	0.87
Xpsmp2208	11	2.60	1.39	0.61

### Population structure

**Fig 1:**
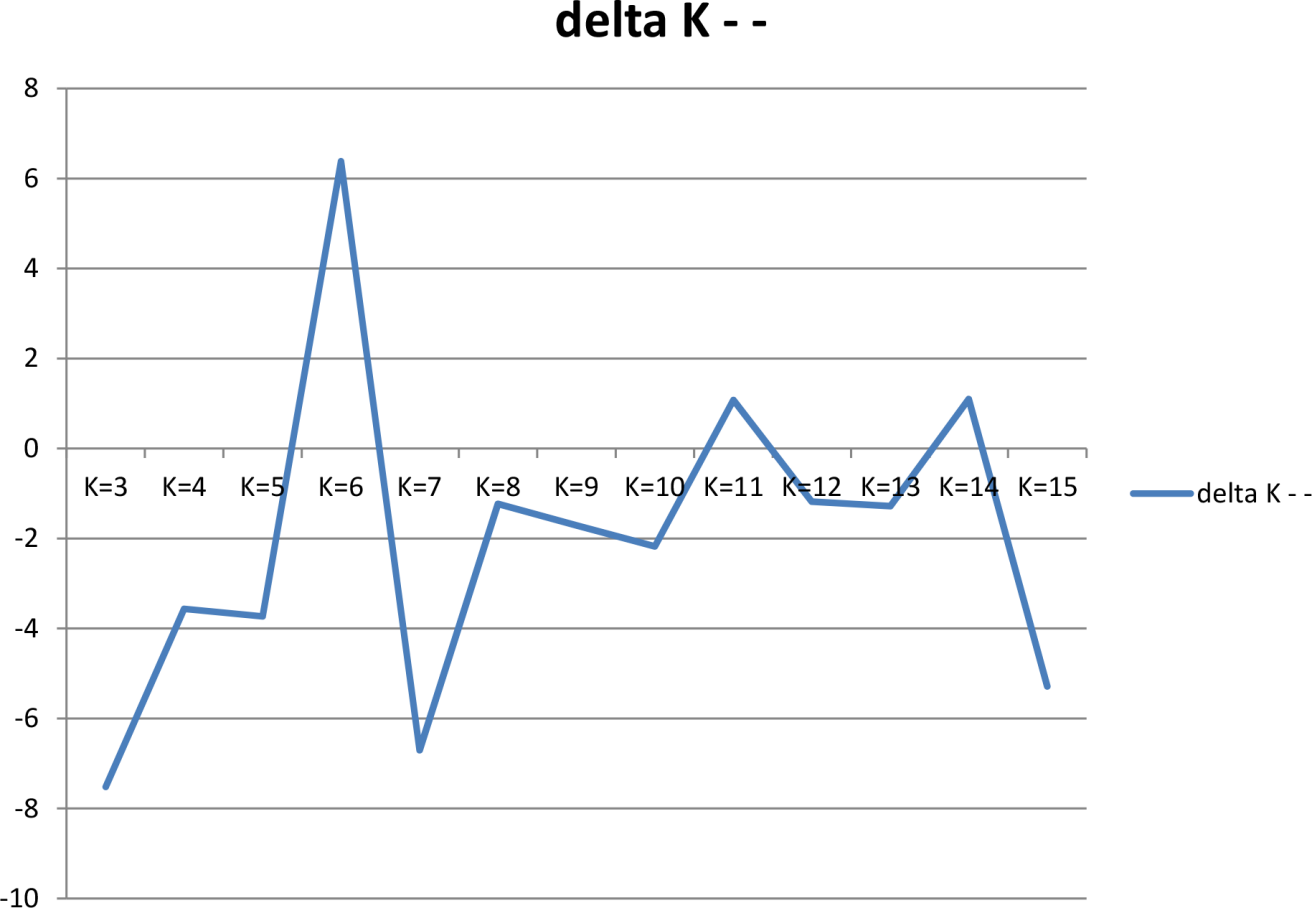
Ad-hoc statistic ∆K for K values ranging from 1 to 15.

**Fig 2:**
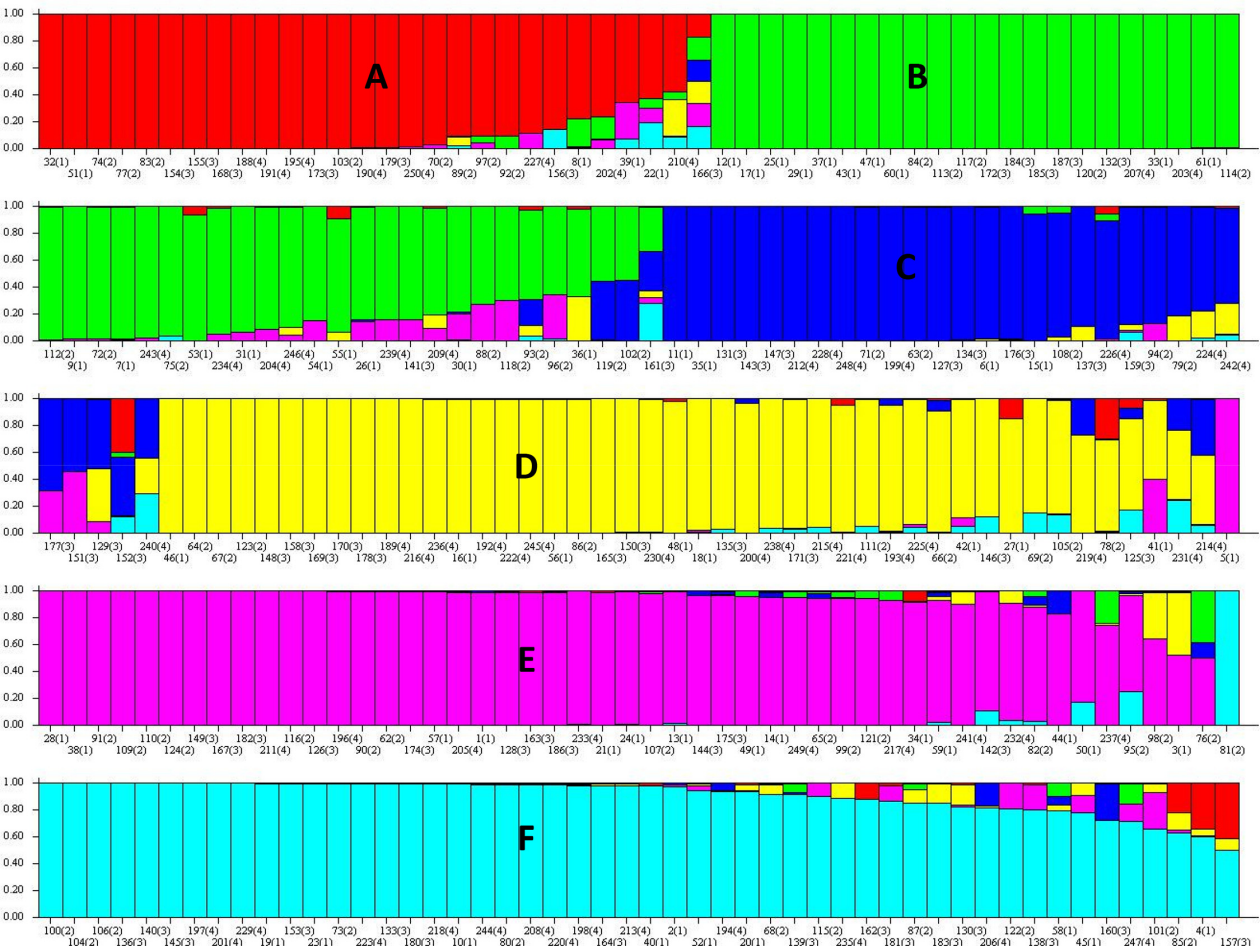
Population structure of PMiGAP based on 37 SSR and CISP markers (K = 6). Each accession is represented by a thin vertical line, which can be partitioned into six colored segments representing estimated membership probabilities (Q) of the individual to the six clusters.

The model-based STRUCTURE analysis was performed for K populations varying from 1 to 15. ∆K showed the highest likelihood at K = 6 (Fig 1) and topologically meaningful clustering was captured at K = 6. Notwithstanding the geographic origins, the six subpopulations (A, B, C, D, E and F) identified by STRUCTURE (Fig 2) corroborated pedigree and/or characteristics of the lines. Population A (*red*) included sixteen accessions from west and east Africa, one accession from southern Africa, one accession from central Africa and two accessions from India. The eight accessions whose source was ICRISAT gene bank were also included in this population. These eight accessions (PRLT 2/89-33, ICMP 451-P6, ICMP 451-P8, ICMP 85410-P7, GS 156, D2 WS, GB 8735 and P1449-3) shared either a pedigree or geographic origin with other west and east African accessions in population A. For instance, both ICMP 451-P6 and ICMP 451-P8 are derived from LCSN 72-1-2-1-1, a selection made in Burkina Faso (West Africa). The accession ICMP 85410-P7 is a derivative of a cross based on a germplasm from east and west Africa. Similarly, PRLT 2/89-33 is an inbred derived from the ICRISAT Bold Seeded Early Composite, based predominantly on Iniadi landrace germplasm from West Africa. Population B (*green*) was a large group comprising of 48 accessions from seemingly diverse geographic origins, including 19 accessions from west Africa, 5 accessions from east Africa, 4 accessions from southern Africa, 2 accessions from central Africa, 9 accessions from India and one accession each from USA, Yemen and South Africa. The six accessions (ICMS 7703, H 77/833-2, 843B, ICMB 89111, IP 8275, GS 154) from ICRISAT gene bank were also part of this large group. As expected, 843B and its derivative ICMB 89111 (bred at ICRISAT using 843B as one of the parents) were grouped together in the population B. The lines with similar traits, but with diverse origins, were generally characteristic of this population. For example, accessions from Yemen (IP 20349) and USA (IP 21155) have characteristic features of productive tillers > 12. The accessions from India and Niger included in this population are either salinity tolerant (IP 6101, IP 3732) or thermo-tolerant (IP 21517, IP 3175). Similarly, three accessions from east and southern Africa (IP 13363, IP 19386, IP 19448) possess characteristic features of thick panicles (>50mm). Two early maturing accessions from west Africa and India (IP 13520, IP 9532) were also part of this population. Population C (*blue*) comprised of 9 accessions from west Africa, 2 accessions each from east and central Africa and 5 accessions from Asia (India and Pakistan). Population C also contained a large (11) number of breeding lines released by ICRISAT for different breeding programs. For example, 81B-P6 and ICMB 90111-P6 are downy mildew (DM) resistant selections which have been widely used as mapping population parental lines (MPPLs). Similarly, LGD 1-B-10, P310-17-B, P1449-2-P1 and WSIL-P8 developed by ICRISAT have been widely used as MPPLs. Another two lines, SOSAT-C88 and Raj 171, released by ICRISAT in west coast of Africa and India, respectively, were also included in this population. Population D (*yellow*) comprised of 44 accessions predominantly from West Africa (18) and 4 accessions each from east, central and southern Africa and one accession each from South Africa and India. This population also included a large number of breeding lines/cultivars developed and released by ICRISAT. The lines Okashana 1 (released in Namibia), GICKV 93191 and WC-C75 (released in India), 863B (drought tolerant; MPPL), IP 18293-P152 (MPPL), ICML 1 and ICML 2 (resistant to ergot) and five others were part of this subpopulation. Population E (*purple*) contained 50 accessions, with 60% of them originating from west (21) and east (8) Africa. The remaining accessions included 3 accessions from central Africa, 9 accessions from India, 1 accession each from Pakistan and USA and 7 breeding lines/cultivars released by ICRISAT. Similar to population B, lines with similar traits yet diverse origins were grouped in this population. For example, salinity tolerant lines from India and Niger (IP 3757, IP 6102) and early maturing (<37 days) lines from Togo and Pakistan (IP 18132, IP 17720) were part of this population. Population F (*turquoise*) was the largest group with 51 accessions of diverse origins including 15 accessions from west Africa, 9 accessions each from east and central Africa, 11 accessions from India, 1 accession each from south Africa and Pakistan, 2 Tifton accessions (Tift 186 and Tift 383) from USA and a few breeding lines released from ICRISAT. Again, lines with similar traits, regardless of geographic origins, were grouped in this population. For example, drought tolerant lines from Uganda and Ghana (IP 8955, IP 9406), lines with sweet stalk from India, Cameroon, Burkina Faso and Nigeria (IP 3471, IP 14439, IP 13817, IP 12128), lines with purplish-black seed colour from Sudan (IP 10759, IP 13324), lines with yellow endosperm from Burkina Faso (IP 15536, IP 15533) and lines with thick panicle from Zimbabwe and Burkina Faso (IP 16403, IP 12845); all were included in population F.

The distance-based principal component analysis (PCA) showed grouping fairly consistent with model-based grouping, except for population D. The population D was merged with populations F and A (results not shown). Genetic diversity parameters calculated for six subpopulations are given in Table 2. The diversity parameters were also estimated in populations from 26 geographic regions (S2 Table). The highest number of observed (5.2) and effective number of alleles (2.9) were observed in the population from India. The expected heterozygosity was also highest in population from India (0.56) followed by Niger and Mali (0.54).When only populations from Africa are compared, the highest number of observed alleles was obtained in population from Burkina Faso (4.60) followed by Niger and Nigeria (4.26 and 4.24, respectively) and the highest effective number of alleles was obtained in population from Niger (2.83) (S2 Table).

**Table 2:** Average observed number of alleles (A), effective number of alleles (A_e_), Shannon’s information index (I), observed and expected heterozygosity (H_o_ and H_e_) and private alleles for the six subpopulations

Subpopulation	A	Ae	I	H_o_	H_e_	Private alleles
A	3.89 (1, 9)	2.37	0.92	0.16	0.51	8
B	4.29 (2, 10)	2.40	0.92	0.28	0.51	11
C	3.62 (1, 9)	2.30	0.88	0.13	0.49	6
D	4.02 (1, 12)	2.40	0.90	0.17	0.49	10
E	4.59 (1, 10)	2.53	0.98	0.21	0.53	18
F	4.94 (2, 12)	2.62	1.00	0.20	0.51	24

### Moisture environments effects

The effect of drought environment was evident on all measured traits (S3 and S4 Tables). The average reduction in grain yield due to drought stress was 24.2 and 29.2% in 2011 and 2012, respectively (S3 and S4 Tables). The similar effects were observed on all yield components, but the absolute reductions varied with trait. Panicle yield (19.5 and 22.1% reduction in 2011 and 2012, respectively), biomass yield (23.2 and 19.7% reduction in 2011 and 2012, respectively), and grain number per square meter (15.5 and 16.1% reduction in 2011 and 2012, respectively) were the traits most affected by drought stress (S3 and S4 Tables). Grain harvest index (GHI) represents the ratio of grain to biomass yield, and panicle harvest index (PHI) is the ratio of grain to total panicle weights. GHI has been advocated as an index of the ability to convert biomass to grain and PHI as a measure of the ability to set and fill grains. We observed almost similar reductions in both GHI and PHI in the late stress conditions but greater in the early stress conditions (S3 Table). These results suggest the greater effect of the early stress (before flowering) on productive tiller number.

### Heritabilities and correlation of traits

**Fig 3:**
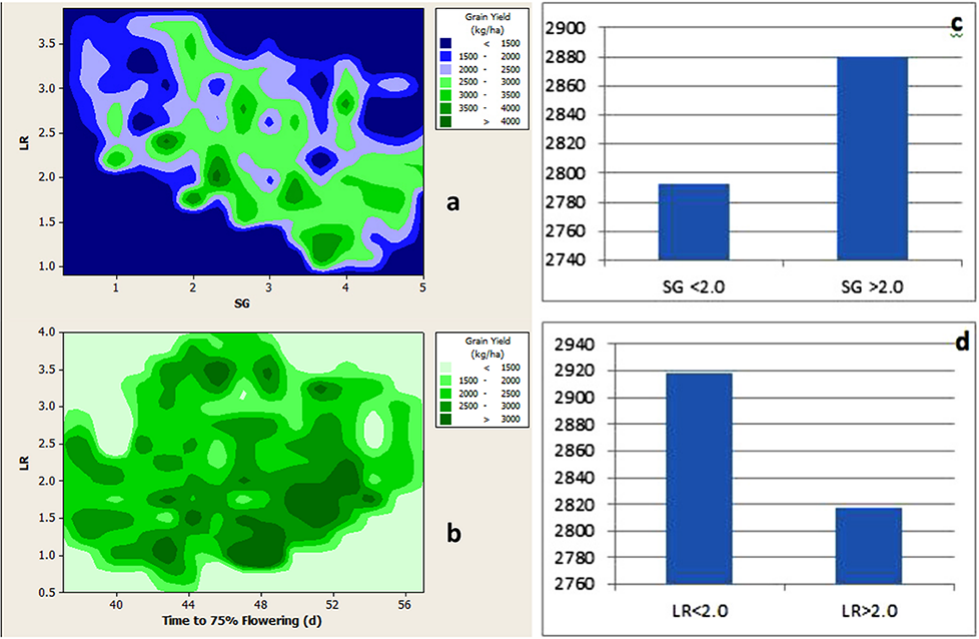
Contour plots of grain yield vs. leaf rolling and stay green (a) and grain yield vs leaf rolling and flowering time under drought stress (b). Effects of stay green (c) and leaf rolling (d) on grain yield under drought stress.

The heritability was generally high (>0.75) or moderately high (0.50–0.75) for all the traits under control conditions (S3 Table). The heritability estimates in early and late stress treatments ranged from 0.60 (BY) to 0.94 (FT) and 0.44 (BY) to 0.94 (FT), respectively. Across environments, heritability estimates for panicle and biomass yield were low (<0.50) and it was moderate for grain yield and panicle harvest index (≥0.50). For remaining traits, these estimates were fairly high (>0.70) (S3 Table). The correlation values among traits under control and drought stress conditions are provided in S5 and S6 Tables. In general, GY and FT were negatively correlated under both control and drought stress conditions but correlation was not significant under drought stress conditions. A significant negative correlation between GY and LR and positive correlation between GY and SG under drought stress conditions in both years (S6 Table) suggest that LR and SG have more effect on GY than FT under terminal drought stress in pearl millet The 3D contour plots confirm this (Fig 3A and 3B). The genotypes having stay green score more than 2.0 showed more grain yield than the genotypes having scores less than 2.0 (Fig 3C). An opposite trend was observed for leaf rolling and grain yield i.e. genotypes having leaf rolling scores more than 2.0 had significantly lesser yield than genotypes having scores less than 2.0 (Fig 3D).

Traits GNPM, PN and GNPP showed significant differences among the six subpopulations under all treatments. Populations C and D had the highest GNPP and GNPM under both control and two drought stress environments while population B had the highest PN under all treatments. Other phenotypic traits were not significantly different among the subpopulations (results not shown).

### Frequency of SNPs in candidate genes and linkage disequilibrium

Previously, we mapped 17 genes as SNP and CISP markers on LG2 in the drought tolerance QTL region [28]. In this study, we genotyped PMiGAP with these gene-based markers and also re-sequenced the genes on a subset of randomly selected 48 genotypes of PMiGAP to identify more SNPs and InDels. The PMiGAP was finally genotyped with 39 SNPs and 7 InDel markers from 17 genes. A total of 251 SNPs with an average of one SNP per 38 bp were identified in 9487 bp sequenced region from 17 candidate genes. The number of SNPs per gene ranged from one in putative acetyl CoA carboxylase, protein phosphatase 1 regulatory subunit SDS22 and PSI reaction center subunit III to 60 in alanine glyoxylate aminotransferase. The average SNP frequency ranged from one SNP per 10 bp in PSI reaction center subunit III to one SNP per 600 bp in acetyl CoA carboxylase (Table 3). The InDel markers ranged from 1 bp (serine carboxypeptidase III precursor) to 20 bp in protein phosphatase 1 regulatory subunit SDS22 (Table 4).

**Table 3:** Frequency of SNPs in genes underlying DT-QTL based on 48 genotypes of PMiGAP.

Gene	Sequenced region	Number of SNPs obtained^a^	Number of SNPs genotyped^b^	Marker name(s)^c^
Serine carboxypeptidase III precursor	710	11	2	Xibmsp07/AP2.1, Xibmsp07/AP2.2
Zinc finger C- × 8-C × 5-C × 3-H type	620	3	2	Xibmsp15/AP1.1, Xibmsp15/AP1.2
Uridylate Kinase	680	21	3	Xibmsp09/AP9.1, Xibmsp09/AP9.2, Xibmsp09/AP9.3
Serine-threonine protein kinase	690	30	2	Xibmsp14/AP3.1, Xibmsp14/AP3.2
Ubiquitin conjugating enzyme	460	4	3	Xibmsp24/AP4.1, Xibmsp24/AP4.2, Xibmsp24/AP4.3
Acyl CoA oxidase	740	14	4	Xibmsp12/AP5.1, Xibmsp12/AP5.2, Xibmsp12/AP5.3, Xibmsp12/AP5.4
Acetyl CoA carboxylase	600	1	1	Xibmsp11/AP6.1
Catalase	270	4	3	Xibmsp26/AP12.1, Xibmsp26/AP12.2, Xibmsp26/AP12.3
Phosphoglycerate Kinase	452	13	4	Xibmcp08/AP8.1, Xibmcp08/AP8.2, Xibmcp08/AP8.3, Xibmcp08/AP8.4
Actin depolymerising factor	650	12		Xibmsp43/AP11.1, Xibmsp43/AP11.2
Chlorophyll A/B binding protein	590	11	2	Xibmcp09/AP10.1, Xibmcp09/AP10.2
Dipeptidyl peptidase IV	695	4	2	Xibmsp60/AP17.1, Xibmsp60/AP17.2
Protein phosphatase 1 regulatory subunit SDS22	500	1	1	Xibmcp11/AP7.1
PHYC	350	4	2	Xibmsp55/AP15.1, Xibmsp55/AP15.2
Alanine glyoxylate aminotransferase	700	60	2	Xibmsp27/AP14.1, Xibmsp27/AP14.2
Photolyase	600	40	3	Xibmsp44/AP18.1, Xibmsp44/AP18.2, Xibmsp44/AP18.3
PSI reaction center subunit III	180	18	1	Xibmsp53/AP16.1

^a^ Total number of SNPs obtained in the sequenced region in 48 randomly selected genotypes from within PMiGAP

^b^ Number of SNPs genotyped in PMiGAP using KASPar genotyping system

^c^ Marker name given to a particular SNP is based on previous nomenclature (28)

**Table 4:** Indel markers designed in the present study.

Gene	Marker name^a^	Forward primer	Reverse primer	Size of InDel
Uridylate kinase	Xibmsp09/AP	TTACATAAGTAATCATGAATCACAAGG	TTGTCCATCTGAGAAGCCAGT	2bp
Serine carboxypeptidase III precursor	Xibmsp07/AP	TTGGGACACGGTAAGGAATG	ATTGGTTCCGTCAAAACTGG	1bp
Serine-threonine protein kinase	Xibmsp14/AP	CAAGGTATCTTCATCTTACAGCAA	TCACGAGTTACAACTCCACTTTT	1bp
Protein phosphatase 1 regulatory subunit SDS22	Xibmcp11/AP	TGAACTTGGTAGCAACGGATT	TCATCAAACATGATTTGGTTCC	20bp
Actin depolymerising factor	Xibmsp43/AP	GCCAGCTACGAGGATTTCAC	GCAAGCACAGATGACAAGGA	2bp
Phosphoglycerate kinase	Xibmcp08/AP	CCGCAGGCACTGAGGTAATA	CGGTTGAAATGTGGCTCATC	2bp

^a^ Marker name given to a particular InDel is based on previous nomenclature (28)

The extent of LD was assessed among all 1,575 pairs of loci (LD calculated for loci mapped on LG2) for all accessions as well as for the six subpopulations separately. Across all accessions, 28% of the total marker pairs were in LD (based on squared correlation coefficient *r* ^*2*^; P<0.01). When LD was calculated within each subpopulation, the frequency of pairs of loci with significant LD (P<0.05) was reduced by more than half.

**Fig 4:**
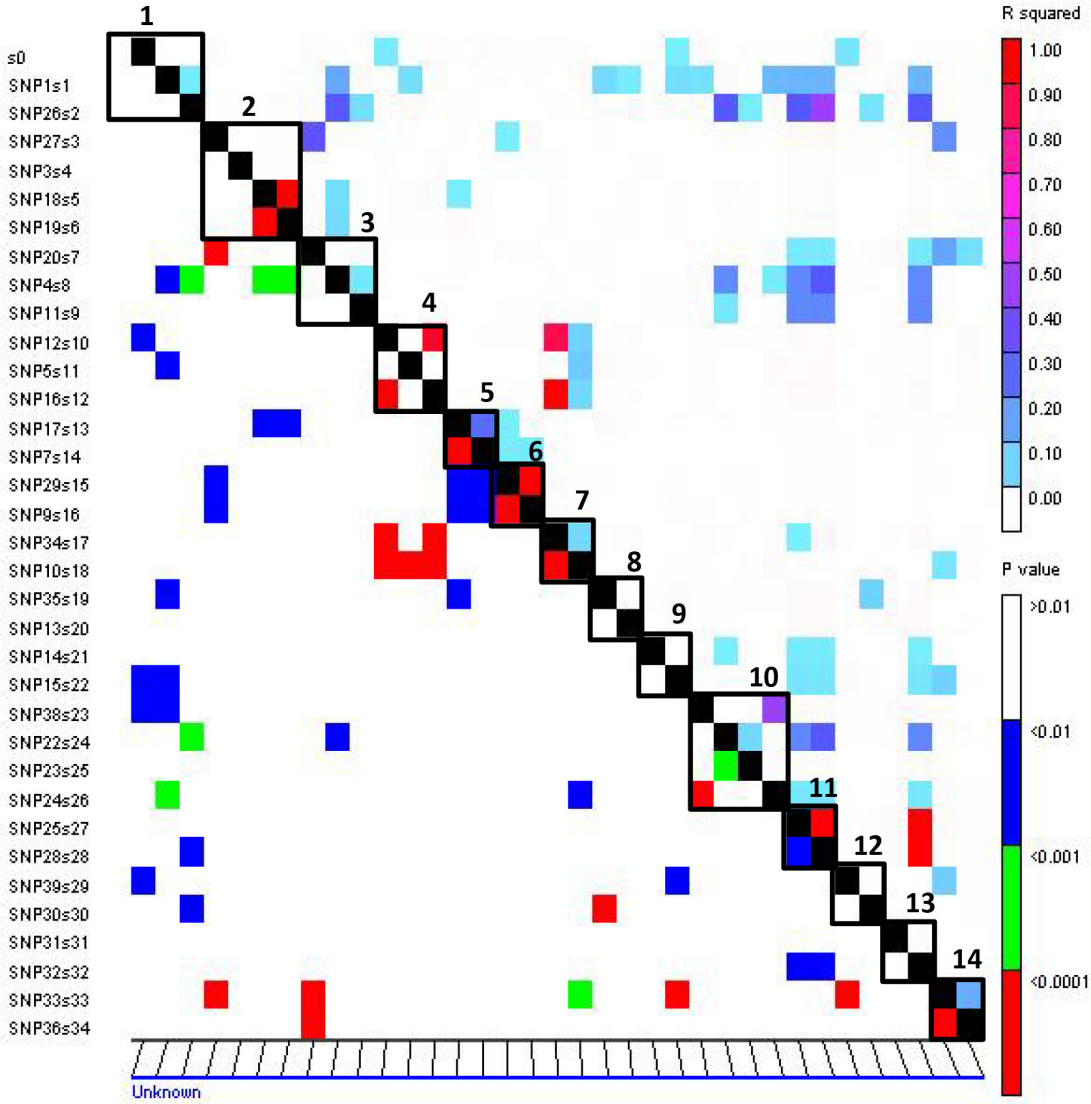
Intragenic linkage disequilibrium in candidate genes. The squared correlation coefficient (*r* ^*2*^) values are denoted by a color scale from white (0.0) to red (1.0) in the upper triangle. The p values ranging from non-significant (0.01; white) to highly significant (<0.0001; red) are shown in the lower triangle. 1. Uridylate kinase 2. Acyl CoA oxidase 3. Zn finger CCCH type 4. Ubiquitin conjugating enzyme 5. Actin depolymerising factor 6. Phytochrome C 7. Dipeptidyl peptidase IV 8. Serine carboxypeptidase 9. Serine/threonine protein kinase 10. Phosphoglycerate kinase 11. Chl a/b binding protein 12. Catalase 13. Alanine glyoxalate aminotransferase and 14. Photolyase.

For candidate genes where multiple SNPs were genotyped across all PMiGAP entries, average intragenic LD (*r* ^*2*^) values were calculated (Fig 4). The average *r* ^*2*^ ranged from 0 in alanine glyoxylate aminotransferase, catalase, serine/threonine protein kinase and serine carboxypeptidase III precursor to 1.0 in putative Chlorophyll a/b binding protein and phytochrome C (Fig 4). The average *r* ^*2*^ in putative Zn finger CCCH type, ubiquitin conjugating enzyme, acyl CoA oxidase, phosphoglycerate kinase, uridylate kinase, actin depolymerizing factor, dipeptidyl peptidase IV and photolyase was 0.02, 0.32, 0.17, 0.10, 0.02, 0.29, 0.09 and 0.18, respectively.

### Association between genes and traits

**Fig 5:**
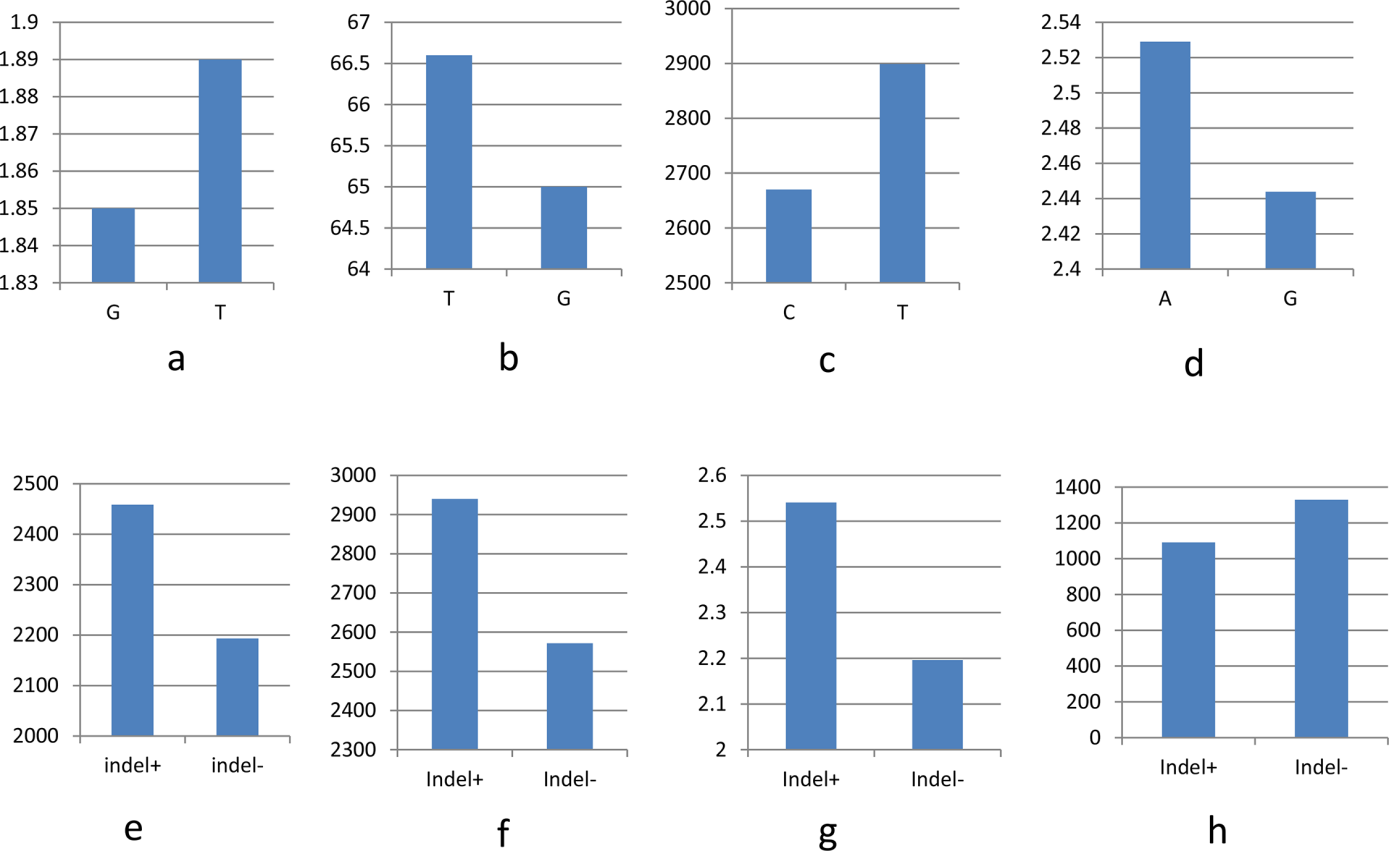
Allele effects of (a) T/G SNPs in putative uridylate kinase gene on tiller per plant under late stress, (b) T/G SNPs in putative serine/threonine protein kinase gene on PHI under late stress, (c) C/T SNPs in acetyl CoA carboxylase gene on grain yield under late stress, (d) A/G SNPs in Zn finger CCCH type on panicle diameter under late stress, (e) InDel in putative chlorophyll a/b binding protein on grain yield under early stress, (f) InDel in putative chlorophyll a/b binding protein on grain yield under late stress, (g) InDel in putative chlorophyll a/b binding protein on stay green under late stress, and (h) InDel in putative phopsphoglycerate kinase on grain number per panicle under early stress.

The two model approaches, general linear model (GLM) and mixed linear model (MLM), were compared for all traits using both Q-matrix and three PCAs in both models. The results were same irrespective of the matrix (Q- or PCA-matrix) chosen in the models. The QQ plots of traits (S1 Fig) suggested that the MLM model is superior at accounting for spurious associations resulting from population structure and/or familial relatedness. Here we present results of only MLM model-based associations. Briefly, out of 39 SNPs and 7 InDels genotyped in 250 genotypes of PMiGAP, we found significant (P<0.05) association for 22 SNPs and 3 InDels from 13 candidate genes with drought tolerance traits. Out of 6 InDel markers developed in the present study (Table 4), we obtained significant associations with two InDel markers, one each in uridylate kinase and phosphoglycerate kinase genes. One InDel marker designed from chlorophyll a/b binding protein gene (*Xibmcp09*) in previous study [28] showed significant association with traits only under drought stress conditions. Table 5 shows SNPs and InDels from 17 genes associated with traits under different environments (irrigated and two drought stress treatments). Fig 5 shows significant allelic effects of various genes on different traits.

**Table 5:** Marker-trait associations in PMiGAP.

Candidate gene	SNP name	Trait	R^2^ (%)	Stress treatment
Serine carboxypeptidase III precursor	Xibmsp07/AP2.1	GHI	3.0	Late
		PHI	3.1	Late
	Xibmsp07/AP2.2	GHI	3.1	Late
		PHI	2.9	Late
		PD	2.8	Late
Zinc finger C- × 8-C × 5-C × 3-H type	Xibmsp15/AP1.1	PD	2.6	Irrigated
		PHI	2.5	Irrigated
		TPP	2.5	Irrigated
		PD	2.0	Late
	Xibmsp15/AP1.2*	TGW**	2.0	Irrigated
		PD	2.5	Late
		TGW**	3.4	Late
Uridylate Kinase	Xibmsp09/AP9.1	GNPP	2.8	Late
	Xibmsp09/AP9.2*	TGW**	2.7	Late
	Xibmsp09/AP9.3*	GY	4.6	Irrigated
		GHI	8.2	Irrigated
		GNPM	3.3	Irrigated
		PD	3.0	Irrigated
		PH	4.8	Irrigated
		PHI**	5.8	Irrigated
		PL	2.9	Irrigated
		PN	4.2	Irrigated
		TPP	38.6	Irrigated
		GHI	3.5	Late
		PN	2.8	Late
		TPP	11.7	Late
	Xibmsp09/AP (InDel)	GHI	2.6	Irrigated
		PN	2.9	Late
Serine-threonine protein kinase	Xibmsp14/AP3.2	GNPM	2.8	Irrigated
		PHI	3.8	Late
Ubiquitin conjugating enzyme	Xibmsp24/AP4.1*	GHI	3.2	Irrigated
		PY	2.7	Irrigated
		GNPP	3.6	Late
		SG**	4.0	Late
		GHI	3.5	Late
	Xibmsp24/AP4.3*	TGW**	3.4	Irrigated
		GHI**	3.0	Late
		GNPP**	3.7	Late
		SG**	3.3	Late
Acyl CoA oxidase	Xibmsp12/AP5.2*	LR**	5.6	Late
		PHI**	3.0	Late
		PN	2.9	Late
		TPP**	2.9	Late
		PHI	3.6	Late
		PN	3.0	Late
		TPP	3.5	Late
		TPP**	4.6	Early
	Xibmsp12/AP5.3 *	LR	2.5	Late
		PH	3.5	Late
		PN	5.9	Late
	Xibmsp12/AP5.4	TPP**	4.1	Late
		GNPP	4.0	Irrigated
		GNPP	2.7	Late
		PL	2.7	Late
		TPP	3.1	Late
		PN	5.3	Late
Acetyl CoA carboxylase	Xibmsp11/AP6.1*	GHI**	6.0	Irrigated
		PHI**	9.0	Irrigated
		PY	3.9	Irrigated
		GY	6.1	Early
		GHI**	3.6	Early
		LR	4.7	Early
		PY	6.6	Early
		GY**	5.3	Late
		GHI**	4.7	Late
		PHI	3.9	Late
		PY	4.1	Late
		GNPP	2.9	Late
		LR	3.7	Late
		PD	5.0	Late
		PN	3.0	Late
		TPP	3.0	Late
Phosphoglycerate kinase	Xibmcp08/AP8.1*	PD	3.0	Early
		SG	3.7	Early
		TGW**	2.2	Late
	Xibmcp08/AP8.2	TGW	3.3	Late
	Xibmcp08/AP8.3	PHI	2.8	Irrigated
		LR	2.4	Late
		PD	2.2	Late
	Xibmcp08/AP(InDel)	GNPP	6.2	Early
		PHI	4.9	Late
Chlorophyll A/B binding protein	Xibmcp09 (InDel) *	GY**	4.2	Early
		TGW**	5.0	Early
		PY	4.3	Early
		GY**	6.5	Late
		GHI	4.8	Late
		PD	4.2	Late
		PHI	4.3	Late
		PY**	5.7	Late
		SG	4.0	Late
		TGW**	5.4	Late
		LR**	5.0	Late
Dipeptidyl peptidase IV	Xibmsp60/AP17.2	GY	3.0	Late
		GNPP	3.3	Late
		SG	3.4	Late
		GHI	3.2	Late
Protein phosphatase 1 regulatory subunit SDS22	Xibmcp11/AP7.1	PY	3.0	Early
		GY	3.0	Late
		LR	3.5	Late
		PHI	3.0	Late
		PY	3.0	Late
PHYC	Xibmsp55/AP15.1*	SG**	3.4	Early
		PD	2.9	Early
		PD	3.2	Irrigated
	Xibmsp55/AP15.2	SG	3.2	Early
		PHI	3.2	Irrigated
		PL	2.8	Late
		PD	3.5	Early
		PHI	2.9	Early
Alanine glyoxylate aminotransferase	Xibmsp27/AP14.1*	PHI	3.4	Irrigated
		PL**	5.6	Irrigated
		PL**	5.0	Early
		LR	3.0	Early
		PD	3.0	Early
		PL**	3.6	Late
		PY	3.0	Late

*Candidate genes-SNPs associated with trait in 2011 and 2012 separately as well as combined data from both years

**Traits for which associations were observed in 2011 and 2012 separately as well as combined data from both years

### Semi-quantitative RT PCR of candidate genes

**Fig 6:**
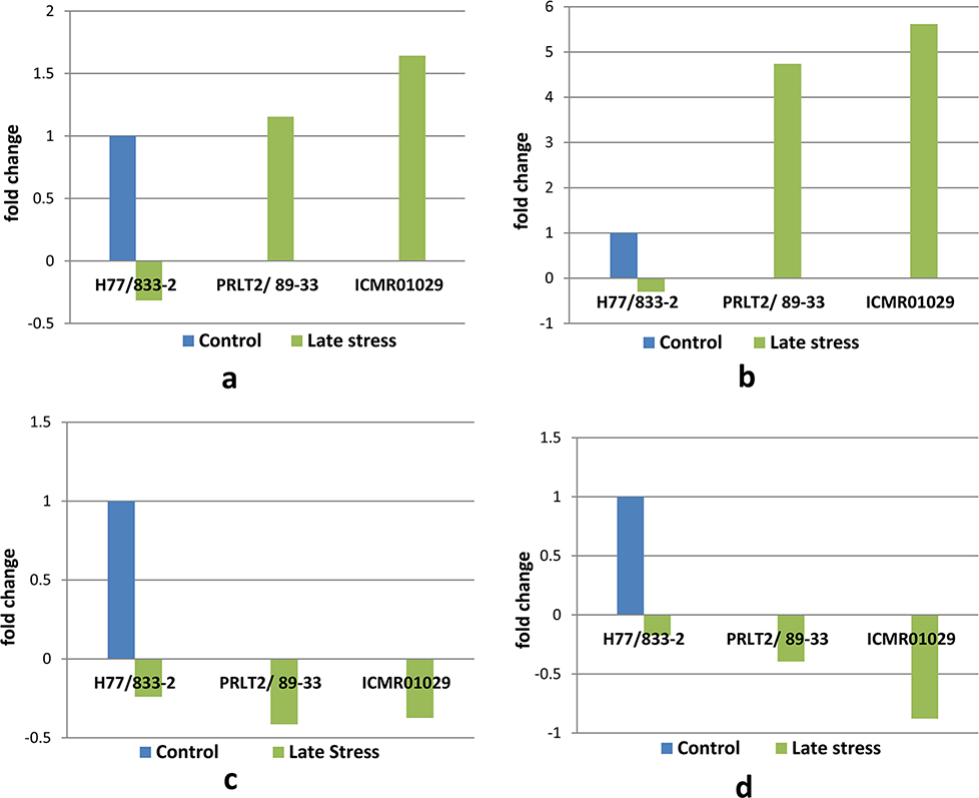
Semi-quantitative PCR of putative genes under terminal drought stress treatment: (a) Zn finger CCCH type, (b) Chlorophyll a/b binding protein, (c) Ubiquitin conjugating enzyme and (d) Serine/threonine protein kinase.

Semi-quantitative PCR was conducted on four candidate gene sequences (putative Zn finger CCCH type, chlorophyll a/b binding protein, ubiquitin conjugating enzyme and serine/threonine protein kinase) using drought tolerant (PRLT 2/89-33) and susceptible (H77/833-2) parents of a mapping population which was used to identify DT-QTL on LG2 [14, 15], and a near isogenic line (NIL) ICMR 1029 having DT-QTL introgressed from PRLT2/89-33. All the four genes showed differential expression between drought tolerant and sensitive parents under drought stress (Fig 6). In drought tolerant parent and NIL, transcript accumulation of putative Zn finger CCCH type and chlorophyll a/b binding protein increased significantly whereas in drought sensitive parent their levels decreased under drought stress (Fig 6). The transcript levels of putative ubiquitin conjugating enzyme and serine/threonine protein kinase decreased by ≥2-fold in tolerant parent and NIL as compared to sensitive parent H77/833-2 (Fig 6).

## Discussion

A pearl millet germplasm association panel (PMiGAP), comprised of 250 landraces, elite cultivars and mapping population parents, collected from a wide geographical range in Africa and Asia, was recently established from the global pearl millet germplasm collection of ICRISAT [27]. The present study represents the first report of genetic diversity in PMiGAP and exploitation of this germplasm panel for association mapping of drought tolerance traits.

Across the 250 PMiGAP entries examined, we obtained a total gene diversity value of 0.54. This value is close to gene diversity estimates obtained previously for a pearl millet world collection from Africa and Asia [38, 39] and higher than gene diversity estimates obtained by Budak *et al*., [40] and Mariac *et al*., [41] for a world-wide collection of pearl millet maintained in Plant Genetic Resources Conservation Unit at University of Georgia and cultivated pearl millet landraces from Niger, respectively. However, the gene diversity estimates in PMiGAP are lower than those obtained for pearl millet landraces derived from West and Central Africa [42]. The lower gene diversity found here, despite having a large representation from West and Central Africa, could be caused by the fact that we used a lot of gene-based CISP (conserved intron spanning primers) markers that were developed previously [28] in addition to SSRs with different repeat motifs. In contrast, Stich *et al*., [42] used a higher proportion of dinucleotide SSR markers, which are more variable than CISPs (produce two alleles at a locus) and SSRs with longer repeat motifs. The average number of alleles (7.0) in the present study was close to those reported previously in pearl millet with SSR markers [38–41] but less than reported by Stich *et al*., [42] for the same reason as described before.

Comparison of genetic diversity parameters, estimated based on twenty-six geographic origins, revealed the highest observed and effective number of alleles in genotypes from India followed by Burkina Faso, Nigeria, Niger and Mali (S2 Table). The expected heterozygosity was also highest in genotypes from India followed by Niger and Mali. Similar trend was also observed for number of private alleles (S2 Table). These results indicate the highest genetic diversity in populations of pearl millet from India followed by West Africa. The results also suggest that pearl millet collections from India can be a useful resource for introducing novel variations into elite germplasm.

The STRUCTURE analysis revealed six sub-populations in PMiGAP (Fig 2). This was consistent with the results of PCA (results not shown). The six groups obtained within PMiGAP did not correspond with origin of country or agro-ecological zone but with pedigrees and/or similar agronomic traits. These results are consistent with previous reports of genetic diversity in pearl millet germplasm from similar geographic areas [38–40, 42]. The present and previous studies, therefore, suggest that genetic diversity in pearl millet has been shaped largely by diversifying human selection rather than geographic origin [42]. The present results also support extensive germplasm exchange among different geographical regions [40].

The candidate gene-based association mapping is a promising approach to bridge the gap between quantitative and molecular genetic approaches for complex trait dissection [43]. Using this approach, polymorphic sites within candidate genes are linked with phenotypic variation using statistical methods to identify causative polymorphisms [43]. In the present study, 17 genes underlying a major drought tolerance (DT) QTL [28] were sequenced to find SNPs (Table 3) and for studying their association with drought tolerance traits in PMiGAP. We obtained a higher SNP frequency in the present study (Table 3) as compared to our previous study [28], which is not unexpected considering the different number of genotypes used for SNP discovery in the two studies. In maize, a contrasting variation in SNP frequency was also reported in two different germplasm sets having different number of accessions [44, 45]. Our present results of SNP frequency are similar to those obtained in many other outcrossing species such as perennial ryegrass [26], maize [45], rye [46] and sugar beet [47].

The linkage disequilibrium measure *r* ^*2*^ varied extremely from gene to gene in the present study (Fig 4). For example, it was 0 in putative alanine glyoxylate aminotransferase, catalase, serine/threonine protein kinase and serine carboxypeptidase III precursor and it was 1.0 in putative putative chlorophyll a/b binding protein and phytochrome C. The *r* ^*2*^ in the remaining genes varied from less than 0.10 (putative Zn finger CCCH type, phosphoglycerate kinase, uridylate kinase and catalase) to upto 0.32 (ubiquitin conjugating enzyme, acyl CoA oxidase, phosphoglycerate kinase, uridylate kinase, actin depolymerizing factor, dipeptidyl peptidase IV and photolyase). These results are not unexpected considering that linkage disequilibrium estimates vary according to the target region in the gene (introns, exons etc.) and number of polymorphic sites [48, 49]. In sorghum and barley, *r* ^*2*^ in the candidate genes (CGs) ranged from 0.024 to 0.21 [25] and 0.0 to 1.0 [50], respectively. The mean *r* ^*2*^ was 0.23 for CGs in our study, which is comparable to *r* ^*2*^ obtained for CGs in sorghum [25] and *Lolium perenne* [51]. Since for most of the genes studied here only a part was sequenced (ranging from 180 to 740 bp for different genes; Table 3), we could not study the decay of linkage disequilibrium along each CG. Full-length sequencing of the genes will become possible in future as whole genome sequencing project reaches completion in pearl millet.

We tested both GLM, MLM models for all traits, and obtained QQ plots to choose the best model for association analysis. It has been reported that directly fitting both Q/PCA (structure) and K (kinship), without testing the model, may overcorrect population structure and familial relatedness for some traits and results in type II error [52]. No significant deviations were observed between the observed and expected values for the traits under different treatments using the MLM model (S1 Fig). Thus, based on QQ plots we found that MLM model is the best for controlling both type I and type II errors. Further, prior to initiating association analysis with CGs, we tested SSR markers underlying DT-QTL for association analysis using Q+K and PCA+K models. We obtained significant associations of *Xpsmp2059*, *Xipes0152*, *Xicmp3056*, *Xipes0117* and *Xipes0218* with many drought tolerance traits (results not shown) which confirms previous results of association of these markers with DT-QTL based on bi-parental fine mapping population [28].

Out of 39 SNPs and 7 InDels from 17 genes genotyped in PMiGAP, significant associations were obtained for 22 SNPs and 3 InDels from 13 genes considering all treatments together (one irrigated and two drought stress treatments) (Table 5). Of these, four genes belong to classes of transcription factors (Zn finger CCCH type), signaling proteins (serine/threonine protein kinase and protein phosphatase 1) and (or) regulatory proteins (ubiquitin conjugating enzyme). Zn finger CCCH type belongs to a large and diverse family of transcription factors members, which play important roles transcriptional regulation, RNA binding and protein-protein interactions [53]. Phosphorylation and dephosphorylation, catalyzed by a variety of protein kinases and phosphatases, respectively, modulate a wide range of biological processes such as the stability and subcellular localization of target proteins, protein-protein interactions, and regulation of plant K^+^ channels in guard and mesophyll cells [54–56]. Similarly, ubiquitin conjugating enzyme is part of ubiquitin proteasome system playing a significant role during senescence in remobilization of amino acids to supply the developing organs elsewhere in the plant. The major function of ubiquitination is to select target proteins for proteasomal degradation. The covalent attachment of ubiquitin to a target protein involves an enzyme cascade mediated by three enzymes, ubiquitin activating enzyme E1, ubiquitin conjugating enzyme E2 and ubiquitin ligase E3 [57]. Many recent reports have described the important roles played by Zn finger CCCH type transcription factors, serine/threonine protein kinases, protein phosphatases and ubiquitin conjugating enzymes in the regulation of plant response and adaptation to multiple abiotic stresses including drought stress [57–63]. However, whether these genes contribute to the natural variation in plant responses to drought stress is not known yet. Once proven, it can provide impetus to initiate gene-based breeding in many crops and speed up the development of stress-tolerant varieties. The present study is the first report of association of these important genes with natural variations in traits under drought stress in a germplasm panel through association analysis (Table 5).

Except for protein phosphatase 1 regulatory subunit SDS22, we obtained significant associations of SNPs in the remaining three genes under both irrigated and drought stress conditions. For example, SNPs *Xibmsp15/AP1*.*1* (A/G) and *Xibmsp15/AP1*.*2* (A/G) in Zn finger CCCH type are associated with panicle diameter (PD) and thousand grain weight (TGW), respectively, under both irrigated and late drought stress conditions. Since both PD and TGW are highly correlated with grain yield, these two SNPs can be used for marker-assisted selection (MAS) in pearl millet for improving yield under both irrigated and water-limited environments. The SNP in serine/threonine protein kinase gene, however, was not associated with the same trait under both irrigated and drought stress conditions (Table 5) i.e. it was associated with grain number per m^2^ (GNPM) under irrigated conditions and with panicle harvest index (PHI) under late stress conditions. Hence, these SNPs can be used for MAS either in specific environment or in combination with other SNPs from genes showing significant associations with GNPM and PHI. Similarly, the SNP in protein phosphatase 1 gene can be used for MAS for grain yield, panicle harvest index and panicle yield under drought stress conditions. For ubiquitin conjugating enzyme, we obtained significantly associated SNPs for grain harvest index, panicle yield and thousand grain weight under irrigated conditions and for stay green, grain number per panicle and grain harvest index under late drought stress conditions. The significant association of SNPs in ubiquitin conjugating enzyme (*UBC*) gene with stay green trait under terminal drought stress confirms the importance of this gene during leaf senescence [57, 64].

We obtained positive correlation of grain yield with stay green under terminal drought stress treatments (Fig 3; S6 Table) which suggests that delayed senescence or stay green phenotype during reproductive and ripening stages is important for genetic improvement of grain yield in pearl millet under drought stress. In addition, a significant negative correlation of leaf rolling with grain yield points to the equal importance of high water extraction via high transpiration for reproductive success under terminal drought stress. Our present results are in agreement with recent findings of Kholova and Vadez [65] in pearl millet who demonstrated that sustained ‘stay green phenotype’ and ‘transpiration’ during grain filling are crucial for grain yield under drought stress. ‘Stay green’ is an important trait reflecting the capacity of the plant to photosynthesize. A strong correlation between the photosynthetic capacity and grain yield has been reported in many cereals including wheat and maize [66, 67]. For example, wheat genotypes with the ability to maintain green leaf area throughout grain filling duration have been suggested as potential candidates for improving yield in arid and semi-arid regions [68]. Also in maize and sorghum, stay green lines have been used in breeding programmes to enhance yield of these important grain crops [69].

Of the genes that underlie DT-QTL, Zn finger CCCH type has been functionally characterized in rice [62, 70]. Transgenic rice overexpressing Zn finger CCCH type exhibited delayed senescence of leaves and retained more photosynthetic activity even under heading and seed-setting stages [62]. In the present study, semi quantitative RT-PCR of putative Zn finger CCCH type and chlorophyll a/b binding protein genes in drought tolerant and sensitive lines exposed to terminal drought stress revealed increased expression of both genes in drought tolerant lines and decreased expression in drought sensitive line (Fig 6) thus suggesting a direct effect of this transcription factor on downstream genes for photosynthesis. These results are similar to that obtained in rice [62, 70] and further confirm the role of Zn finger CCCH type as a negative regulator of leaf senescence. Furthermore, it has been demonstrated that the potential targets of this transcription factor are key genes required to promote the leaf senescence program such as receptor-like kinases, which function as key components in the perception of environmental signals and subsequent phosphorylation cascades [70]. The semi-quantitative RT-PCR carried out for putative serine/threonine protein kinase gene in drought tolerant and sensitive lines under drought stress, as expected, showed a 2–4 fold decreased expression in tolerant lines as compared to the sensitive genotype (Fig 6). In addition, a 2-fold decreased expression of putative *UBC* gene was observed in drought tolerant lines as compared to the sensitive genotype. These results indicate that key signaling enzymes and ubiquitin proteasome system are repressed in pearl millet under terminal drought stress in order to keep the photosynthetic machinery intact.

We obtained significant association of an InDel in putative chlorophyll a/b binding protein gene with grain yield, thousand grain weight and panicle yield under both early and late drought stress conditions and with grain harvest index, panicle diameter, panicle harvest index, stay green and leaf rolling under late stress conditions (Table 5). Fig 5 (e, f and g) shows clearly that the genotypes carrying this InDel show stay green phenotype and correspondingly higher grain yield under terminal drought stress conditions. The chloroplast located light-harvesting chlorophyll a/b-binding proteins (LHCP) collect and transfer light energy to photosynthetic reaction centers [71, 72]. Recently, allelic variations in *LHCP* gene have also been reported to be associated with plant height, spike length, number of grains per spike, thousand grain weight, flag leaf area and leaf color under well-watered conditions in barley [72]. The significant association of an InDel in *LHCP* gene with grain yield and yield components, and stay green traits under terminal drought stress is an important finding of the present study. This InDel can be used for MAS in pearl millet to select high yielding genotypes having ‘stay green’ phenotype under terminal drought stress.

The role of phytochromes in sensing moisture conditions has been reported. For example, *PhyE* has been associated with local adaptation to dry environments like alpine habitats [73]. *PhyB* is reported to be involved in gas exchange rates [74, 75] and in the regulation of abscisic acid (ABA) metabolism [76]. Recently, Boggs *et al*., [77] demonstrated the roles of *PhyA*, *PhyB* and *PhyE* in influencing stomatal conductance plasticity via ABA regulation under drought stress. In the present study, we found significant associations of two SNPs in *PhyC* with many traits including stay green, panicle diameter, panicle harvest index and panicle length under both well-watered and drought stress conditions. Previously, *PhyC* has been shown to be associated with agronomic traits in pearl millet under well-watered conditions [78].

Acetyl CoA carboxylase (*ACoC*) is a key enzyme in fatty acid biosynthesis. Fatty acid biosynthesis involves the cyclic condensation of two-carbon units derived from acetyl-coA [79]. The first committed step in the pathway is the synthesis of malonyl-coA from acetyl-coA and CO_2_ by the enzyme *ACoC*. The tight regulation of *ACoC* controls the overall rate of fatty acid synthesis. It has been shown in barley that ceasing activity of *ACoC* inhibits growth and development of seedlings as there is not enough fatty acids to contribute to membrane structures [80]. We genotyped a single SNP in *ACoC* gene in PMiGAP entries and found significant association of this SNP with many traits (Table 5), most importantly with grain yield, grain harvest index and panicle yield under all conditions i.e. fully-irrigated non-stressed condition as well as early and late drought stress treatments. This SNP marker can be used for MAS in pearl millet for improving yield under both irrigated and terminal drought stress conditions (Table 5). Further, under late drought stress condition this SNP was associated with many other yield components thus indicating the importance of this gene as the stress progresses.

Like the ubiquitin proteasome system, the prolyl oligopeptidase family plays a significant role in storage protein mobilization during defense system activity and senescence. The prolyl oligopeptidase family of serine proteases includes four members; prolyl oligopeptidase (POP), dipeptidyl peptidase IV (DPPIV), oligopeptidase B (OPB), and acylaminoacyl peptidase (ACPH). Of these, POP has been demonstrated to confer drought stress tolerance [81]. The roles of other members of this family are not known yet. In the present study, we obtained evidence of the role of another member of this family, DPPIV, under drought stress. We identified significant associations of DPPIV with grain yield, grain number per panicle, stay green and grain harvest index under late drought stress conditions (Table 5).

Similar to the results demonstrated in this study, many genes have been reported to be associated with drought tolerance using association mapping [82–84] thus reinforcing the complexity of the trait. Of all the marker-trait associations obtained in the present study, 7 associations from putative genes Zn finger CCCH type, uridylate kinase, ubiquitin conjugating enzyme, acetyl CoA carboxylase and phytochrome C were common under irrigated and one (or both) of the drought stress treatments indicating the potential of these associations to be applicable for MAS (Table 5). Of these, the most significant was the SNP in putative acetyl CoA carboxylase gene, which was significantly associated with grain yield, grain harvest index and panicle yield under all water stress treatments (Table 5). We did not obtain any significant association of putative catalase gene with any of the traits in our study. These results support those obtained by [85] in pearl millet suggesting that the anti-oxidant machinery does not play a direct causal role on the terminal drought tolerance of pearl millet that is conferred by DT-QTL.

Correlation among traits is commonly used by breeders for either simultaneous improvement of correlated traits or for reducing undesirable side effects when improving only one of the correlated traits. The negative correlation is undesirable by breeders specifically when the goal is to simultaneously increase the trait values of two negatively correlated characters. Many examples can be cited for such negatively correlated traits. For example, a negative correlation exists between grain yield and protein content in maize [86], durum wheat [87], and soybean [88]. In the same way, positive correlations can also be undesirable. For example, a positive correlation between high oil and beta-glucan concentrations in oats is highly undesirable by breeders targeting high oil yield with less beta-glucan [89]. On the positive side, such positive correlations have been successfully exploited to improve difficult traits or traits involving costly measurements. For instance, in maize the anthesis-silking interval is positively correlated with grain yield and it has been used as an indirect selection criterion for improving grain yield under drought stress [90].

The two major reasons for such genetic trait correlations at the level of QTL are pleiotropy and/or close linkage of genes. In the present study, we chose a major DT-QTL on LG2 that is basically a drought-tolerance grain yield QTL with QTLs for grain harvest index, panicle harvest index, biomass yield and other yield components, and stay green/delayed senescence co-mapping to the same interval [14–16, 28, 65]. The results of the present study helped to understand the cause of co-mapping of these traits in this interval or alternatively the underlying genetic cause of these multiple-trait associations. We conclude that both tight linkage of genes and intragenic linkage of quantitative trait polymorphisms are the molecular basis of these multiple-trait associations. For instance, *UBC*, *LHCP* and *PhyC* are the key regulatory and downstream genes in the DT-QTL interval found to be associated with the stay green trait in the present study (Table 5). Association analysis revealed unambiguously that each of the SNPs (or InDel) in each of these three genes is also significantly associated with grain yield and/or yield components. From a plant breeding perspective, these results are desirable and encouraging. For example, a functional marker derived from InDel in *LHCP*, affecting stay green and grain yield simultaneously (Fig 5), can be fixed in the breeding materials.

## Conclusion

In the present study, we investigated the genetic diversity and structure in PMiGAP and explored its potential for association analysis. We obtained high genetic diversity in PMiGAP and a moderate genetic structure, which is ideal for conducting association analysis. Using MLM model, many significant marker-trait associations were obtained some of which can be used as functional markers for selecting high yielding plants under irrigated and drought stress conditions. Particularly, a SNP in putative acetyl CoA carboxylase gene and an InDel in putative chlorophyll a/b binding protein gene were the most important associations, worth using for MAS in pearl millet. Results also confirmed that PMiGAP is a useful panel for association mapping.

## Supporting Information

S1 Fig QQ plots of GY, GHI, PHI and GNPM using (a) MLM and (b) GLM models. GY = Grain yield, GHI = Grain harvest index, PHI = Panicle harvest index, GNPM = Grain number per panicle, MLM = Mixed linear model, GLM = General linear model.
(TIF)

S1 Table List of pearl millet accessions used in the study, their origin and characteristics, and estimated fraction of the accession’s genome that originates from six inferred subpopulations (subpopulations A, B, C, D, E and F). (PDF)

S2 Table Genetic diversity parameters in populations from 26 geographic origins. (PDF)

S3 Table Mean and heritability of the traits. (PDF)

S4 Table Reductions in growth and yield parameters due to drought stress in 2011 and 2012. (PDF)

S5 Table Correlation among traits under control conditions. (PDF)

S6 Table Correlation among traits under drought stress conditions. (PDF)
