# Supplementary figures and images for: Exploring Potential of Pearl Millet Germplasm Association Panel for Association Mapping of Drought Tolerance Traits
Source: PLoS One. 2015 May 13;10(5):e0122165. doi: 10.1371/journal.pone.0122165 (PMC4430295; doi:10.1371/journal.pone.0122165)

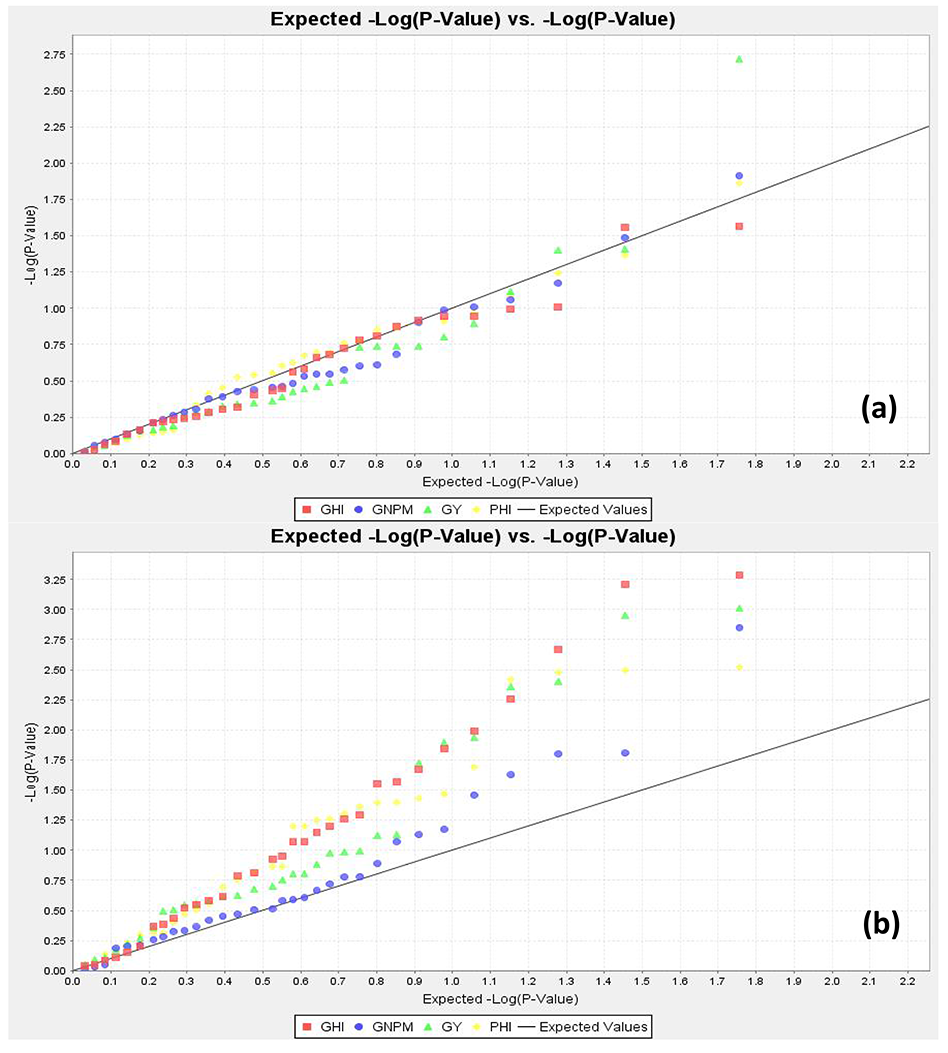

Supplement: S1 Fig — GY = Grain yield, GHI = Grain harvest index, PHI = Panicle harvest index, GNPM = Grain number per panicle, MLM = Mixed linear model, GLM = General linear model. (TIF) [file pone.0122165.s001.tif]
