# Supplementary material for: Exploring Potential of Pearl Millet Germplasm Association Panel for Association Mapping of Drought Tolerance Traits
Source: PLoS One. 2015 May 13;10(5):e0122165. doi: 10.1371/journal.pone.0122165 (PMC4430295; doi:10.1371/journal.pone.0122165)
Supplement: S1 Table — (PDF) [file pone.0122165.s002.pdf]

S1 Table. List of pearl millet accessions used in the study, their origin and characteristics, and estimated fraction of the accession's genome that originates from six inferred subpopulations (subpopulations A, B, C, D, E and F)

S.no.	Accession code	IP no.	Alternate accession identifier	Origin/Source	Comment	Subpop. A	Subpop. B	Subpop. C	Subpop. D	Subpop. E	Subpop. F	Inferred subpop.
1	AP88	IP 10820	Acc 615	Sudan		0	0	0.0105	0	0.9895	0	E*
2	AP84	IP 10964		Kenya		0	0.0001	0.0134	0.0001	0.0127	0.9737	F
3	AP81	IP 3098		India		0.0067	0	0.0005	0.4668	0.5216	0.0044	D/E**
4	AP90	IP 8949	P 3254; PL 73; Yoei	Togo	Resistant to rust	0.3418	0	0	0.0495	0.0043	0.6044	A/F
5	AP77	IP 13154	Maiwa local 2-1	Niger		0	0	0	0	1	0	E
6	AP76	IP 9407		Ghana		0.0009	0	0.9796	0.0194	0	0.0001	C
7	AP95	IP 20349		Yemen	Productive tillers (>12)	0.0013	0.9799	0.01	0.0037	0.0049	0.0002	B
8	AP92	IP 11229		Zimbabwe	Large seed (>18 g)	0.7736	0.2103	0	0.0022	0.0139	0	A/B
9	AP74	IP 13370	Msungu-mukufi	Tanzania		0	0.9844	0	0	0.0156	0	B
10	AP83	IP 11353	CVP 298	Burkina Faso		0.002	0	0	0.001	0.0038	0.9932	F
11	AP91	IP 8181	IP 338-1	ICRISAT-patencheru		0	0	1	0	0	0	C
12	AP85	IP 18157	Souna	Mali		0	1	0	0	0	0	B
13	AP89	IP 13964		Zimbabwe		0	0.0001	0	0.0072	0.9753	0.0174	E
14	AP86	IP 4965	Serere 53	Uganda		0.0008	0.0097	0.0363	0	0.9532	0	E
15	AP97	ICMB 90111-P6		ICRISAT-Patencheru		0.0001	0.0527	0.9457	0	0.0015	0	C
16	AP66	IP 17690		Togo		0.0009	0	0	0.9991	0	0	D
17	AP70	IP 6101	P 936	Niger	Salinity tolerant	0	1	0	0	0	0	B
18	AP69	IP 10488		Zimbabwe		0.0001	0	0	0.9782	0.012	0.0097	D
19	AP71	IP 13149	P 932-1	Niger	Salinity tolerant	0.0006	0	0	0	0	0.9994	F
20	AP72	IP 15533		Burkina Faso	Yellow endosperm	0.0115	0.0002	0.0007	0.038	0.0127	0.9369	F
21	AP68	IP 10140	P 5510; Chowuguile	Mali		0.012	0	0.0008	0	0.9848	0.0024	E
**S.No.**	**Accession code**	**IP no.**		**Origin/Source**	**Comment**	**Subpop. A**	**Subpop. B**	**Subpop. C**	**Subpop. D**	**Subpop. E**	**Subpop. F**	**Inferred subpop.**
22	AP67	IP 3890		India		0.626	0.0724	0	0.0002	0.1044	0.197	A/F
23	AP64	IP 11677	100; Dukhun	Sudan		0.0017	0	0	0	0	0.9983	F

24	AP65	IP 11984		Nigeria	Long panicle_Postrainy (>100 cm)	0.0009	0	0.0004	0.0009	0.9836	0.0142	E
25	AP58	IP 3175	Chadi	India	Thermo tolerant	0	1	0	0	0	0	B
26	AP55	W 504-1-P1		India	Elite inbred	0.0006	0.8405	0.0128	0.0012	0.144	0.0009	B
27	AP57	IP 22455	ICMV-IS 89305	ICRISAT	Seed color_Yellow	0.1455	0	0	0.8545	0	0	D
28	AP46	IP 16096		India		0	0	0.0001	0	0.9999	0	E
29	AP49	IP 6060	P 175; Tein	Central African Republic		0.0001	0.9999	0	0	0	0	B
30	AP47	IP 7633	S 195	India		0.0002	0.7834	0.0111	0	0.1966	0.0087	B/E
31	AP60	IP 19386		Namibia	Thick panicles_Rainy (>54 mm)	0	0.9286	0	0	0.0688	0.0026	B
32	AP42	IP 6112	P 949	Niger	Salinity tolerant	1	0	0	0	0	0	A
33	AP33	IP 10811	Acc 604	Sudan	AP33	0	0.9969	0.0031	0	0	0	B
34	AP41	IP 3757		India	Salinity tolerant	0.0773	0.0016	0	0	0.9211	0	E
35	AP38	IP 18147		Pakistan	Early_Rainy (<37 days)	0	0	1	0	0	0	C
36	AP43	IP 19448		Namibia	Thick panicles_Rainy (>54 mm)	0.0187	0.6509	0	0.3236	0.0017	0.0051	B/D
37	AP37	ICMS 7703	ICMS 7703	ICRISAT-patencheru		0	1	0	0	0	0	B
38	AP35	IP 11577	P 6041; Yan nane	Burkina Faso		0	0	0	0	1	0	E
39	AP36	GB 8735	GB 8735	ICRISAT	ISC, released in WCA	0.6557	0	0	0.0002	0.2696	0.0745	A/E
40	AP44	ICML 22		Chad		0.0189	0.0005	0	0	0	0.9806	F
**S.No.**	**Accession code**	**IP no.**		**Origin/Source**	**Comment**	**Subpop. A**	**Subpop. B**	**Subpop. C**	**Subpop. D**	**Subpop. E**	**Subpop. F**	**Inferred subpop.**
41	AP45		Okashana 1 (ICMV 88908)	ICRISAT-Patancheru	Released in Namibia	0.0113	0.003	0	0.579	0.4047	0.002	D/E
42	AP31	IP 9446		Ghana	Overall plant aspect (=9)	0.0008	0	0	0.8776	0.0645	0.0571	D
43	AP32	IP 21155	Tift 23DAS	USA	Productive tillers	0.0001	0.9999	0	0	0	0	B

					(>12)							
44	AP24	IP 11211	PSR 49-1	India		0	0	0.1702	0.0008	0.825	0.004	E/C
45	AP22	IP 6745		Malawi		0.0001	0	0	0.0894	0.1313	0.7792	F
46	AP21	IP 6769		Malawi		0	0	0.0001	0.9998	0.0001	0	D
47	AP25	IP 10705	CMM 540; Haini	Mali		0	1	0	0	0	0	B
48	AP26	IP 22274	GICKV 93191 (ICMP 93191)	India	Released in MP	0.0145	0	0	0.9855	0	0	D
49	AP27	IP 22272	AIMP 92901	India	Released in MH	0	0.0415	0	0	0.9585	0	E
50	AP17	IP 18132		Pakistan	Early_Rainy (<37 days)	0	0.0001	0	0.0002	0.8245	0.1752	E/F
51	AP15		PRLT 2	ICRISAT-Patancheru	BSEC pollinator	1	0	0	0	0	0	A
52	AP16	IP 4020		India	Early_Postrainy (<40 days)	0	0.0009	0	0.0166	0.0385	0.944	F
53	AP11	IP 13971		Zimbabwe	AP11	0.0577	0.9397	0.0017	0.0005	0.0004	0	B
54	AP8	IP 8767		Botswana	Bristle length_Long	0	0.8455	0	0	0.1545	0	B
55	AP9	IP 12298		Nigeria	Long panicle_Postrainy (>100 cm)	0.087	0.8426	0.0022	0.0652	0.002	0.001	B
56	AP10		863B-P2	ICRISAT-patencheru	Mapping pop parent	0.0006	0	0	0.9977	0.0017	0	D
57	AP4		ICTP 8203	ICRISAT-Patancheru	Released in India	0	0.0062	0	0	0.9938	0	E
58	AP3	IP 4542		India		0	0.0953	0.0689	0.0379	0.0003	0.7976	F
59	AP6	IP 17720		Togo	Early_Rainy (<37 days)	0.001	0.0091	0.0287	0.0256	0.9128	0.0228	E
**S.No.**	**Accession code**	**IP no.**		**Origin/Source**	**Comment**	**Subpop. A**	**Subpop. B**	**Subpop. C**	**Subpop. D**	**Subpop. E**	**Subpop. F**	**Inferred subpop.**
60	AP2		H 77/833-2-P5(NT)	ICRISAT-patencheru	Mapping pop parent, drought sensitive	0.0001	0.9999	0	0	0	0	B
61	AP1		843B	ICRISAT-patencheru		0	0.9916	0	0	0.0084	0	B
62	AP196	IP 6102	P 938	Niger	Salinity tolerant	0.0003	0	0	0.0046	0.9951	0	E
63	AP195	IP 3616	Periya	India	Salinity tolerant	0.0016	0	0.9945	0.0005	0.0026	0.0008	C
64	AP184	IP 18389		Namibia		0	0	0	1	0	0	D

65	AP194	IP 9824	Mexioera	Mozambique	Endosperm texture_Mostly corneous	0.006	0.0125	0.0367	0	0.9444	0.0004	E
66	AP204	IP 18293	P152			0.0095	0.0004	0.0829	0.8983	0.006	0.0029	D
67	AP203	IP 22419	ICML 1; ICMPE 13-6-27	ICRISAT	Resistant to ergot	0	0	0	1	0	0	D
68	AP155	IP 11765	Arnold 2141	South Africa		0	0.0018	0.0077	0.0754	0	0.9151	F
69	AP156	IP 6869	Acc 106	Kenya		0	0	0	0.8497	0	0.1503	D
70	AP159	IP 10085	P 5439;Tiotioni	Mali		0.9712	0	0.0002	0.0001	0.0247	0.0038	A
71	AP160	IP 10394	IC 46963	India		0	0.0007	0.9986	0	0	0.0007	C
72	AP172	IP 21517	Sadore local	Niger	Thermo tolerant	0.0006	0.9821	0	0	0.0171	0.0002	B
73	AP164		ICMV-IS 92222	ICRISAT	ISC, released in WCA	0.0006	0.0013	0	0.001	0	0.9971	F
74	AP161	IP 12839		Botswana		1	0	0	0	0	0	A
75	AP168	IP 3732		India	Salinity tolerant	0	0.9618	0.0004	0	0	0.0378	B
76	AP167	IP 8210	IP 1739 L-1	ICRISAT	Drought tolerant	0	0.3799	0.1197	0	0.5004	0	B/E
77	AP158	IP 7108		India		0.9999	0.0001	0	0	0	0	A
78	AP157	IP 5272	D 258	Niger		0.293	0.0009	0.0088	0.6815	0.0052	0.0106	A/D
79	AP166	IP 7910	D 89 C-1-1	Niger	Brown midrib	0	0	0.809	0.1908	0	0.0002	C/D
80	AP169	IP 9391		Ghana	Seed color_Ivory	0	0	0	0.0017	0.0093	0.989	F
81	AP170	IP 3471	Podi cumbu	India	Sweet stalk	0	0	0	0	0	1	F
82	AP148	IP 3636	Mochai	India	Productive tillers (>12)	0.0001	0.0379	0.0684	0.0105	0.8504	0.0327	E
83	AP135	IP 9710	PI 287043	Nigeria		1	0	0	0	0	0	A
84	AP137	IP 3122		India		0	1	0	0	0	0	B
**S.No.**	**Accession code**	**IP no.**		**Origin/Source**	**Comment**	**Subpop. A**	**Subpop. B**	**Subpop. C**	**Subpop. D**	**Subpop. E**	**Subpop. F**	**Inferred subpop.**
85	AP136	IP 6037	P 152; Tein	Central African Republic		0.2147	0	0.0001	0.1326	0.0234	0.6292	F/A
86	AP140	IP 5713	45-349	Nigeria		0.0024	0	0	0.9975	0.0001	0	D
87	AP138	IP 6146	P 42; Mefie	Cameroon		0.0059	0.0373	0.0003	0.1001	0.0021	0.8543	F
88	AP142		ICMB 89111-P2	ICRISAT-patencheru	Mapping pop parent	0	0.7219	0	0	0.2781	0	B/E
89	AP141	IP 10953	BM 8	kenya		0.9066	0.002	0	0.0661	0	0.0253	A
90	AP131	IP 6460	P 482	Mali		0	0	0	0.0033	0.9967	0	E
91	AP52		ICMV 221ICMV 88904	ICRISAT		0.0001	0	0	0	0.9999	0	E
92	AP130	IP 4979	700164	Nigeria		0.9017	0.0977	0.0005	0.0001	0	0	A

93	AP132	IP 9351		Ghana		0.0237	0.6663	0.1946	0.0749	0.0016	0.0389	B/C
94	AP133	IP 10539	CSM 51	Senegal		0	0.006	0.8592	0	0.1348	0	C
95	AP153		Tift 238D1-P158	USA	D1 dwarf	0.0001	0.0042	0.0112	0.0172	0.7163	0.251	E/F
96	AP152	IP 11310	CVP 152	Burkina Faso		0.0001	0.6554	0	0	0.3276	0.0169	B/E
97	AP154	IP 15344		India	Productive tillers (>12)	0.9046	0.0499	0	0	0.0454	0.0001	A
98	AP120	IP 12058		Nigeria	Productive tillers (<2)	0.0008	0.0001	0.0118	0.3415	0.6458	0	E/D
99	AP118	IP 19626	C 90-133; Zongo	Niger	Long panicle_rainy (>100 cm)	0.0031	0.0456	0	0.008	0.943	0.0003	E
100	AP108		Tift 186	USA,	elite tall forage pollinator	0.0001	0	0	0	0	0.9999	F
101	AP126	IP 16403	Halale	Zimbabwe	Thick panicle_Postrainy (>50 mm)	0.003	0.0002	0	0.068	0.2702	0.6586	F/E
102	AP128	IP 2058		Nigeria	Total tillers (<2)	0.0004	0.5461	0.4532	0	0.0003	0	B/C
103	AP99	IP 10486		Zimbabwe		0.9979	0.0018	0	0	0.0003	0	A
104	AP125	IP 12845	Saouga local 2-1	Burkina Faso	Thick panicle_Postrainy	0	0	0	0	0.0002	0.9998	F
**S.No.**	**Accession code**	**IP no.**		**Origin/Source**	**Comment**	**Subpop. A**	**Subpop. B**	**Subpop. C**	**Subpop. D**	**Subpop. E**	**Subpop. F**	**Inferred subpop.**
105	AP104	IP 5695	45-327	Nigeria		0	0.0073	0.0042	0.8436	0.0066	0.1383	D
106	AP101	IP 4962	Serere 50	Uganda		0	0	0	0	0	1	F
107	AP124	IP 15320		India	Sweet stalk	0.0023	0.0148	0.0036	0	0.978	0.0013	E
108	AP105	IP 12768	SM 3646	ICRISAT-patencheru (India)	AP105	0.0001	0.048	0.9202	0.0277	0	0.004	C
109	AP114	IP 8280	Souna 57-1	ICRISAT	Glossy (gl3)	0	0	0.0002	0.0001	0.9997	0	E
110	AP106	IP 6179	P 75; Midougen	Cameroon		0	0	0.0001	0.0001	0.9998	0	E
111	AP100	IP 3557	Mathuravellai	India		0.0018	0	0	0.9469	0.0007	0.0506	D
112	AP116	IP 10271	EC 134725	Nigeria	Long panicle_rainy (>100 cm)	0.0029	0.987	0	0	0.0092	0.0009	B
113	AP109	IP 8761		Botswana	Bristle length_Long	0.0001	0.9995	0.0001	0	0.0003	0	B
114	AP112	IP 9532		Ghana	Early_Rainy (<37 days)	0	0.9908	0	0.0009	0.0066	0.0017	B

115	AP110	IP 8955	P 3271; PL 80; Ignie	Togo	Drought tolerant	0	0	0	0	0.0933	0.9067	F
116	AP117	IP 12322	Gero	Nigeria	Long panicle_Postrainy (>100 cm)	0.0003	0.0001	0	0	0.999	0.0006	E
117	AP119	IP 3564	Umi	India	Productive tillers (<2)	0	1	0	0	0	0	B
118	AP102	IP 8344		India		0	0.6955	0	0	0.3045	0	B/E
119	AP113	IP 8275	IP 1881-1	ICRISAT-patencheru	Glossy (gl1)	0.0004	0.5498	0.4385	0	0.0113	0	B/C
120	AP115	IP 10379	EC 134857	Nigeria	Long panicle_Postrainy (>100 cm)	0	0.9982	0.0018	0	0	0	B
121	AP107	IP 7095		India		0.0001	0.0574	0	0	0.9425	0	E
122	AP111	IP 9406		Ghana	Drought tolerant	0	0	0	0	0.1863	0.8137	F/E
123	AP79	IP 17554		Togo		0	0	0	1	0	0	D
124	AP75	IP 11311	CVP 159	Burkina Faso		0	0.0001	0	0	0.9999	0	E
125	AP258	IP 8166	GS 148	ICRISAT	Purple plant	0.0685	0.0003	0.0793	0.6769	0.0003	0.1747	D/F
**S.No.**	**Accession code**	**IP no.**		**Origin/Source**	**Comment**	**Subpop. A**	**Subpop. B**	**Subpop. C**	**Subpop. D**	**Subpop. E**	**Subpop. F**	**Inferred subpop.**
126	AP246	IP 9969	1769	Zambia		0	0	0	0	0.9987	0.0013	E
127	AP245	IP 10543	CMM 374; Sanio	Mali		0.006	0	0.994	0	0	0	C
128	AP256	IP 7930	IP 5300-2; Maiwa local 1-2	ICRISAT	Brown midrib, Densely hairy	0.0025	0.0074	0	0	0.9901	0	E
129	AP255	WSIL-P8		ICRISAT-patencheru	Mapping pop parent	0.0004	0.0055	0.5144	0.3914	0.0881	0.0002	C/D
130	AP252	IP 18090		Pakistan		0.0086	0	0	0.1502	0.0131	0.8281	F
131	AP253	P 1449-2-P1			Mapping pop parent	0.0001	0	0.9999	0	0	0	C
132	AP257	IP 4927	Souna D2	Senegal	Overall plant aspect (=9)	0	0.9982	0	0	0.0018	0	B
133	AP247	IP 6882	Acc 124	Kenya		0	0	0	0.0038	0	0.9962	F
134	AP259	IP 5253	D 202	Niger	Salinity tolerant	0.0001	0	0.9865	0.0134	0	0	C
135	AP263	IP 18500		Namibia		0	0.0002	0	0.9666	0	0.0332	D
136	AP262	IP 8863		Zambia		0	0.0001	0	0	0	0.9999	F
137	AP264	IP 11346	CVP 278	Burkina Faso	Large seed (>18 g)	0	0	0.8924	0.1071	0	0.0005	C
138	AP250	IP 8069	DS 279	India		0.0086	0.0033	0	0.0015	0.1855	0.8011	F/E
139	AP238	IP 4828	Bareilly 10	India		0.0041	0.0649	0.0134	0.0024	0.0003	0.9149	F
140	AP242	IP 19584	C 90-90; Enele	Niger	Salinity tolerant	0	0	0	0	0	1	F

141	AP240	IP 5207	D 110	Niger		0	0.8375	0	0	0.1625	0	B/E
142	AP239	IP 11275	CVP 9	Burkina Faso		0.0005	0.0004	0	0.0048	0.881	0.1133	E
143	AP241	SOSAT-C88		ICRISAT	ISC, released in WCA	0	0	0.9999	0	0	0.0001	C
144	AP243	IP 6417	P 424; Sanio barbu	Mali		0	0	0.0287	0	0.9713	0	E
145	AP234	IP 14439	Mouri	Cameroon	Sweet stalk	0.0002	0	0	0	0	0.9998	F
146	AP233	IP 9651	PI 286865	Nigeria	Seed yield potential (High)	0	0	0	0.8738	0	0.1262	D
147	AP235	LGD 1-B-10			Mapping pop parent	0	0	1	0	0	0	C
148	AP222	IP 5131	D 235; Zongo	Niger		0	0	0.0002	0.9998	0	0	D
149	AP227	IP 8187	IP 2695-1	ICRISAT	Purple; Long bristles	0.0001	0.0001	0	0	0.9998	0	E
150	AP225	WC-C75		ICRISAT	Released in India	0.0004	0	0	0.989	0.0103	0.0003	D
**S.No.**	**Accession code**	**IP no.**		**Origin/Source**	**Comment**	**Subpop. A**	**Subpop. B**	**Subpop. C**	**Subpop. D**	**Subpop. E**	**Subpop. F**	**Inferred subpop.**
151	AP224	81B-P6		ICRISAT-patencheru	Mapping pop parent	0.0001	0	0.5364	0.0036	0.4564	0.0035	C/E
152	AP228	IP 13927		Zimbabwe	Sweet stalk	0.3984	0.0329	0.4401	0.0005	0.0063	0.1218	A/C
153	AP223	IP 12967		Malawi		0.0011	0	0	0	0	0.9989	F
154	AP230	ICMP 451-P8		ICRISAT-Patancheru	mapping pop parent downy mildew donor	1	0	0	0	0	0	A
155	AP231	ICMP 85410-P7		ICRISAT-Patancheru	mapping pop parent	1	0	0	0	0	0	A
156	AP229	IP 15872	P 15	Tanzania		0.852	0	0.0011	0	0.0003	0.1466	A
157	AP208	IP 9971	1806	Zambia		0.4105	0	0.0004	0.0881	0	0.501	F/A
158	AP211	IP 22420	ICML 2; ICMPE 13-6-30	ICRISAT	Resistant to ergot	0	0	0	1	0	0	D
159	AP209	IP 8074	GS 8	ICRISAT		0.0002	0.0033	0.8747	0.037	0.0153	0.0695	C
160	AP212	Tift 383		USA,	elite d2 dwarf forage pollinator	0.0034	0	0.269	0.0002	0	0.7274	F/C
161	AP216	IP 10632	CMM 465; Haini beri	Mali	Seed yield potential (High)	0.0008	0.329	0.2979	0.0466	0.0434	0.2823	B/C/F
162	AP217	IP 13817	CVP 230	Burkina Faso	Sweet stalk	0.1213	0	0	0	0	0.8787	F
163	AP205	IP 13180	No. 2-1	Nigeria		0.0096	0.0001	0.0002	0	0.9901	0	E
164	AP213	IP 7953	IP 6191-1; P 87-1	ICRISAT	Narrow leaves; Densely hairy	0.0009	0	0	0.0164	0	0.9827	F
165	AP215	IP 10701	CMM 536; Haini	Mali	Seed color_brown	0	0	0.0001	0.9975	0.002	0.0004	D

166	AP210	IP 21169	P 1449-3	ICRISAT	Resistant to rust	0.1712	0.1651	0.1624	0.1616	0.1712	0.1685	A/B/C/D/E/F
167	AP218	IP 8129	GS 112	ICRISAT	Thin panicle_Rainy (<11 mm)	0	0	0	0	1	0	E
168	AP221	ICMP 451-P6		ICRISAT-Patancheru	mapping pop parent downy mildew donor	1	0	0	0	0	0	A
169	AP220	IP 11593	P 6062; Da M'Lare	Burkina Faso		0.0001	0	0	0.9999	0	0	D
**S.No.**	**Accession code**	**IP no.**		**Origin/Source**	**Comment**	**Subpop. A**	**Subpop. B**	**Subpop. C**	**Subpop. D**	**Subpop. E**	**Subpop. F**	**Inferred subpop.**
170	AP219	IP 10471		Zimbabwe		0.0001	0	0	0.9999	0	0	D
171	AP176	IP 7952	IP 6578-1; Kolala local 7-1	ICRISAT		0	0	0.0021	0.9608	0.0033	0.0338	D
172	AP192	IP 9282	P 3279; Ignari	Togo	Drought tolerant	0.0001	0.9999	0	0	0	0	B
173	AP198	IP 17611		Togo	Seed yield potential (High)	0.9989	0	0	0.0009	0	0.0002	A
174	AP185	IP 7886	J 1357-1	India		0	0.0049	0	0	0.995	0.0001	E
175	AP189		ICMV-IS 89305	ICRISAT	ISC, released in WCA	0.0003	0.0068	0.0153	0.0134	0.9611	0.0031	E
176	AP191		Raj 171 (ICMV 85404RBC-IC 9)	ICRISAT-Patancheru	Released in India	0	0	0.9794	0.0116	0.009	0	C
177	AP188	IP 13384	Serere 2A-1	Uganda		0	0	0.6854	0	0.3135	0.0011	C/E
178	AP202	IP 13344	Acc 736-1	Sudan	Total tillers (<2)	0	0	0	1	0	0	D
179	AP178	IP 16638		Zimbabwe		0.9882	0	0.0023	0	0.004	0.0055	A
180	AP180	IP 8972	P 3369; PL 97; Olikon	Togo		0.0001	0	0	0.0015	0.0029	0.9955	F
181	AP200	IP 7967	IP 6342-1; P 337-2	ICRISAT	Thick stems	0.0174	0	0	0.0001	0.1185	0.864	F
182	AP177	IP 5441	P 2731; Zongo	Niger		0	0.0001	0	0	0.9998	0.0001	E
183	AP186	IP 16289		Zimbabwe		0.0001	0.0037	0.0008	0.1424	0.003	0.85	F
184	AP187	IP 6682		Malawi		0	1	0	0	0	0	B
185	AP175	IP 3138		India		0	0.9997	0.0003	0	0	0	B
186	AP182	IP 11584	P 6050; Sanio	Burkina Faso		0.0043	0	0	0.0039	0.9897	0.0021	E
187	AP201	IP 8172	GS 154	ICRISAT-patencheru	Top leaves yellow	0	0.9992	0	0.0001	0.0007	0	B
188	AP343	IP 11218		Zimbabwe		1	0	0	0	0	0	A

189	AP342	IP 18412		Namibia		0	0	0	1	0	0	D
190	AP346	IP 8174	GS 156	ICRISAT-Patancheru	Top leaves yellow	0.9909	0	0.0091	0	0	0	A
191	AP345	IP 6110	P 946	Niger	Salinity tolerant	1	0	0	0	0	0	A
192	AP332	IP 5438	P 2727; Guerguera	Niger		0	0.0001	0.0011	0.9988	0	0	D
193	AP334	IP 11378	CVP 409	Burkina Faso	Large seed (>18 g)	0.0001	0	0.0439	0.9413	0.0071	0.0076	D
**S.No.**	**Accession code**	**IP no.**		**Origin/Source**	**Comment**	**Subpop. A**	**Subpop. B**	**Subpop. C**	**Subpop. D**	**Subpop. E**	**Subpop. F**	**Inferred subpop.**
194	AP325	IP 13608	Kala	India		0	0.0004	0.0554	0.0042	0	0.94	F
195	AP328	IP 8647	Dukhun	Sudan		1	0	0	0	0	0	A
196	AP327	IP 7364		Tanzania		0	0.0001	0.0002	0.0005	0.9988	0.0004	E
197	AP336	IP 10759	Acc 509	Sudan	Seed color_Purplish black	0	0	0	0	0	1	F
198	AP337	IP 15536		Burkina Faso	Yellow endosperm	0.0019	0.0099	0.0011	0.0016	0.0008	0.9847	F
199	AP330	IP 6125	P 15	Cameroon		0	0	0.9989	0	0.0004	0.0007	C
200	AP331	IP 14624		Cameroon		0	0	0.0346	0.9649	0	0.0005	D
201	AP333	IP 12020	Dauro	Nigeria		0.0004	0	0	0	0	0.9996	F
202	AP324	IP 13290	CSM 27-1	Senegal		0.7644	0.1619	0.0003	0.0051	0.0682	0.0001	A/B
203	AP329	IP 11763	Arnold 2131	South Africa		0	0.9971	0.0012	0	0	0.0017	B
204	AP322	IP 13520	Bhilodi	India	Early_Postrainy (<40 days)	0.0038	0.9062	0	0	0.0877	0.0023	B
205	AP335	IP 14398		Cameroon	Large seed (>18 g)	0.0004	0.0049	0.002	0.0016	0.9911	0	E
206	AP317	IP 12128		Nigeria	Sweet stalk	0	0	0.1705	0.0085	0.0002	0.8208	F/C
207	AP316	IP 14311		Cameroon		0	0.9981	0.0008	0	0.0003	0.0008	B
208	AP318	IP 15857		Tanzania		0.0013	0.0001	0	0.0073	0.0034	0.9879	F
209	AP320	IP 12364		Nigeria	Long panicle_Postrainy (>100 cm)	0.0115	0.7905	0	0.0997	0.0906	0.0077	B
210	AP315	IP 18292	D2 WS Gl. yellow	ICRISAT-Patancheru	Resistant to downy mildew	0.5741	0.0588	0	0.2689	0.0074	0.0908	A/D
211	AP309	IP 13840		Burkina Faso		0	0.0001	0	0	0.9999	0	E
212	AP310	IP 5931	P 1538/Sl. 305; Souna	Senegal	Endosperm texture_Mostly corneous	0	0	1	0	0	0	C
213	AP311	IP 4378		India	Overall plant aspect (=9)	0.0005	0	0	0.0161	0.0003	0.9831	F

214	AP304	IP 11929	Belenguine	Sierra Leone	Very late_rainy (>151 days)	0	0.0023	0.4156	0.5114	0.011	0.0597	D/C
215	AP299	IP 10945	Acc 816	Sudan		0	0	0	0.9522	0.0004	0.0474	D
**S.No.**	**Accession code**	**IP no.**		**Origin/Source**	**Comment**	**Subpop. A**	**Subpop. B**	**Subpop. C**	**Subpop. D**	**Subpop. E**	**Subpop. F**	**Inferred subpop.**
216	AP301	IP 7941	IP 5447-1; P 2737-1	ICRISAT	Purple plant	0	0	0	0.9997	0	0.0003	D
217	AP303	IP 17150		Zimbabwe	Thick panicle_Postrainy (>50 mm)	0	0.0679	0.0003	0	0.9318	0	E
218	AP298	IP 13459	Pitta ganti	India		0.0063	0	0	0	0	0.9937	F
219	AP302	IP 8182	IP 406-B-1	ICRISAT	Seed color_Purple	0	0	0.2694	0.7279	0.0007	0.002	D/C
220	AP305	IP 14849		Cameroon		0.0009	0	0	0.0077	0.0047	0.9867	F
221	AP306	IP 5900	P 1505/Sl. 228; Souna	Senegal		0.043	0.0002	0	0.9488	0.0008	0.0072	D
222	AP308	IP 19334		Namibia	Thick panicles_Rainy (>54 mm)	0	0	0.0011	0.9988	0.0001	0	D
223	AP307	IP 3110		India	Productive tillers (>12)	0.0003	0.0002	0	0	0.0025	0.997	F
224	AP296	IP 7536	K 46	India		0	0.0031	0.7706	0.2045	0	0.0218	C/D
225	AP297	IP 12395		South Africa		0.0006	0.0021	0.0012	0.9295	0.021	0.0456	D
226	AP295	IP 14418	Mouri	Cameroon		0.0572	0.0455	0.8818	0	0.0142	0.0013	C
227	AP289	IP 17493		Togo		0.8808	0.0022	0	0.001	0.1115	0.0045	A
228	AP288	IP 6415	P 422; Souna	Mali		0	0	0.9998	0	0.0002	0	C
229	AP290	IP 16120		India	Seed yield potential (High)	0	0	0.0001	0	0.0002	0.9997	F
230	AP292	IP 5560	P 2857-1; Bakin hiri	Niger	Thick panicles_Rainy (>54 mm)	0	0	0.0007	0.9888	0.0001	0.0104	D
231	AP291	IP 12138	Maiwa	Nigeria		0	0	0.2343	0.5154	0.0043	0.246	C/D/F
232	AP294	IP 15512		Burkina Faso	Seed color_brown	0.0001	0	0.0001	0.0913	0.868	0.0405	E
233	AP280	IP 10579	CMM 410; Souna	Mali	Overall plant aspect (=9)	0	0	0	0	0.9855	0.0145	E
234	AP269	IP 17099		Zimbabwe		0.0129	0.9333	0	0.0011	0.0526	0.0001	B
235	AP281	IP 13324	Acc 9-1	Sudan	Seed color_Purplish black	0	0	0	0.1139	0	0.8861	F

S.No.	Accession code	IP no.		Origin/Source	Comment	Subpop. A	Subpop. B	Subpop. C	Subpop. D	Subpop. E	Subpop. F	Inferred subpop.
236	AP276	IP 19405	TCD 9; Arkoum	Chad		0	0.0005	0	0.9995	0	0	D
237	AP268	IP 17125		Zimbabwe		0.0002	0.2394	0.0005	0.0119	0.748	0	B/E
238	AP266	IP 5031	700482	Nigeria		0	0	0	0.9615	0	0.0385	D
239	AP267	IP 11358	CVP 311	Burkina Faso		0	0.8395	0	0.0014	0.1591	0	B
240	AP275	IP 7660	S 395	India		0	0.0003	0.4391	0.2668	0	0.2938	C/D/F
241	AP273	IP 15553		Burkina Faso		0	0.0055	0	0.0904	0.904	0.0001	E
242	AP271	IP 18168	Sounari	Mali		0.0091	0	0.7078	0.2308	0.0061	0.0462	C/D
243	AP283	IP 13363		Tanzania	Thick panicle_Postrainy (>50 mm)	0	0.9751	0	0	0.0249	0	B
244	AP265	IP 6310	P 286	Mali		0	0.0006	0.0007	0.0025	0.003	0.9932	F
245	AP278	IP 20679	Maiwa	Nigeria	Long panicle_rainy (>100 cm)	0	0	0.0014	0.9986	0	0	D
246	AP286	IP 10339	EC 134799	Nigeria		0.0016	0.8956	0.0001	0.0556	0.0468	0.0003	B
247	AP282	IP 3201		India	Thermo tolerant	0.0063	0.145	0.0047	0.0003	0.1256	0.7181	F
248	AP254	P 310-17-B		ICRISAT-patencheru	Mapping pop parent	0.0001	0	0.9999	0	0	0	C
249	AP248	IP 14148		Zimbabwe		0.0006	0.0461	0	0.0003	0.9529	0.0001	E
250	AP244	IP 4952	Serere 41	Uganda	AP244	0.9821	0	0.003	0.0004	0.0145	0	A

*When an accession has q > 0.600 for a subpopulation, the accession is assigned that particular subpopulation

** When an accession has q > 0.160 for two or more subpopulations, the accession is assigned into mixed subpopulation
