# Supplementary material for: Exploring Potential of Pearl Millet Germplasm Association Panel for Association Mapping of Drought Tolerance Traits
Source: PLoS One. 2015 May 13;10(5):e0122165. doi: 10.1371/journal.pone.0122165 (PMC4430295; doi:10.1371/journal.pone.0122165)
Supplement: S2 Table — (PDF) [file pone.0122165.s003.pdf]

S2 Table. Genetic diversity parameters in populations from 26 geographic origins

Population	Na	Na Freq. >= 5%	Ne	I	No. Private Alleles	No. LComm Alleles (<=25%)	No. LComm Alleles (<=50%)	He	uHe
India	5.293	3.146	2.906	1.095	0.341	1.366	2.244	0.561	0.569
Mali	4.122	3.220	2.758	1.032	0.220	0.732	1.366	0.547	0.570
BurkinaFaso	4.463	3.390	2.723	1.024	0.195	0.878	1.634	0.530	0.547
Nigeria	4.244	3.585	2.708	1.005	0.171	0.756	1.537	0.531	0.545
Niger	4.268	3.707	2.830	1.043	0.146	0.951	1.610	0.548	0.565
Sudan	3.561	3.561	2.692	0.984	0.122	0.512	1.024	0.531	0.569
Ghana	2.951	2.951	2.375	0.807	0.073	0.366	0.829	0.448	0.492
Zimbabwe	3.805	3.146	2.545	0.939	0.073	0.585	1.268	0.508	0.526
Kenya	2.317	2.317	2.076	0.652	0.049	0.341	0.610	0.393	0.450
Senegal	2.976	2.976	2.411	0.842	0.049	0.341	0.732	0.483	0.537
Zambia	2.293	2.293	2.043	0.667	0.049	0.293	0.439	0.413	0.498
Cameroon	3.439	3.439	2.549	0.937	0.024	0.488	1.024	0.522	0.554
Central African Republic	1.927	1.927	1.821	0.550	0.024	0.098	0.244	0.366	0.488
Malawi	2.585	2.585	2.148	0.746	0.024	0.341	0.683	0.445	0.509
Mozambique	1.049	1.049	1.049	0.051	0.024	0.024	0.073	0.037	0.073
Pakistan	2.220	2.220	1.991	0.664	0.024	0.268	0.585	0.424	0.516
Tanzania	2.878	2.878	2.318	0.836	0.024	0.317	0.780	0.492	0.549
Togo	3.317	3.317	2.448	0.884	0.024	0.463	1.098	0.485	0.516
USA	1.927	1.927	1.724	0.518	0.024	0.098	0.293	0.335	0.447
Botswana	1.976	1.976	1.801	0.552	0.000	0.049	0.244	0.360	0.434
Chad	1.610	1.610	1.592	0.411	0.000	0.146	0.268	0.293	0.390
Namibia	2.878	2.878	2.286	0.827	0.000	0.268	0.683	0.481	0.527
SierraLeone	1.195	1.195	1.195	0.135	0.000	0.098	0.244	0.098	0.195
SouthAfrica	2.244	2.244	1.860	0.627	0.000	0.220	0.439	0.389	0.476
Uganda	2.488	2.488	2.057	0.751	0.000	0.268	0.585	0.468	0.542
Yemen	1.220	1.220	1.220	0.169	0.000	0.000	0.049	0.122	0.244

Na = No. of Different Alleles

Na (Freq >= 5%) = No. of Different Alleles with a Frequency >= 5%

Ne = No. of Effective Alleles = 1 / (Sum pi^2)

I = Shannon's Information Index = -1* Sum (pi * Ln (pi))

No. Private Alleles = No. of Alleles Unique to a Single Population

No. LComm Alleles (<=25%) = No. of Locally Common Alleles (Freq. >= 5%) Found in 25% or Fewer Populations

No. LComm Alleles (<=50%) = No. of Locally Common Alleles (Freq. >= 5%) Found in 50% or Fewer Populations

He = Expected Heterozygosity = 1 - Sum pi^2

uHe = Unbiased Expected Heterozygosity = (2N / (2N-1)) * He
