# Supplementary material for: Exploring Potential of Pearl Millet Germplasm Association Panel for Association Mapping of Drought Tolerance Traits
Source: PLoS One. 2015 May 13;10(5):e0122165. doi: 10.1371/journal.pone.0122165 (PMC4430295; doi:10.1371/journal.pone.0122165)
Supplement: S3 Table — (PDF) [file pone.0122165.s004.pdf]

S3 Table. Means and heritability of the traits

Trait	Year	Mean ± Std. dev.			h^2			h^2
		Control	Early stress	Late stress	Control	Early stress	Late stress	All environments
GY	2011	3461.88 ± 600.55	2391.27 ± 480.50	2856.32 ± 561.18	0.78	0.72	0.70	0.50
	2012	3158.62 ± 647.77	1909.80 ± 539.68	2571.07 ± 724.92				
PY	2011	4892.66 ± 764.07	3608.94 ± 561.48	4260.19 ± 636.53	0.68	0.66	0.61	0.46
	2012	4548.47 ± 781.60	3190.59 ± 767.68	3910.83 ± 869.88				
PHI	2011	70.75 ± 5.84	64.90 ± 5.15	66.78 ± 6.46	0.55	0.62	0.62	0.56
	2012	69.09 ± 4.77	59.36 ± 5.38	64.87 ± 6.12				
FT	2011	46.13 ± 4.78	45.27 ± 4.06	45.51 ± 4.59	0.94	0.94	0.94	0.94
	2012	51.35 ± 5.04	50.47 ± 4.47	50.27 ± 4.42				
PH	2011	150.43 ± 17.24	158.08 ± 17.68	155.42 ± 17.59	0.90	0.82	0.78	0.84
	2012	156.62 ± 21.80	165.30 ± 24.41	161.22 ± 23.48				
PL	2011	23.10 ± 3.45	22.31 ± 3.25	22.42± 3.02	0.88	0.90	0.87	0.88
	2012	22.15 ± 3.36	22.70 ± 3.42	22.23± 3.45				
PD	2011	2.40 ± 0.29	2.42 ± 0.23	2.43 ± 0.24	0.68	0.73	0.74	0.71
	2012	2.53 ± 0.28	2.33 ± 0.24	2.39 ± 0.27				
PN	2011	279.93 ± 74.11	243.04 ± 61.33	273.37±68.79	0.77	0.80	0.73	0.77
	2012	265.85 ± 65.16	248.74 ± 59.65	261.28±60.38				
TPP	2011	1.94 ± 0.53	1.80 ± 0.46	1.94 ± 0.51	0.71	0.79	0.69	0.74
	2012	1.95 ±0.52	1.68 ± 0.44	1.76 ± 0.41				
BY	2011	8653.99 ± 1160.89	6239.02 ± 861.16	7045.64 ± 950.55	0.52	0.60	0.44	0.40
	2012	8493.12 ± 1200.26	6302.18 ± 1445.50	7389.71 ± 1503.92				
GHI	2011	0.40 ±0.05	0.37 ± 0.05	0.39 ± 0.06	0.87	0.74	0.82	0.79
	2012	0.37 ± 0.06	0.30 ± 0.05	0.32 ± 0.06				
TGW	2011	10.20 ± 1.36	8.56 ± 1.47	9.72 ± 1.44	0.91	0.83	0.84	0.79
	2012	9.38 ± 1.14	7.22 ± 1.19	8.53 ± 1.30				
GNPP	2011	1313.42 ± 411.64	1258.28 ± 450.63	1159.46 ± 378.47	0.87	0.88	0.85	0.87
	2012	1339.20 ± 367.60	1122.83 ± 370.80	1221.74 ± 420.12				

GNPM	2011	34526.31 ± 7511.41	28555.63± 6682.21	29793.26 ± 6488.12	0.83	0.78	0.75	0.76
	2012	34134.27 ± 8092.72	26879.72 ± 7777.60	30514.2 ± 8964.19				

GY, Grain Yield; PY, Panicle yield; PHI, Panicle harvest Index; FT, Flowering time; PH, Plant height; PL, Panicle length; PD, Panicle diameter; PNPH, Panicle number; TPP, Tiller per plant; BY, Biomass yield; GHI, Grain harvest index; TGW, Thousand grain weight; GNPP, Grain number per panicle; GNPM, Grain number per M^2
