# Supplementary material for: Exploring Potential of Pearl Millet Germplasm Association Panel for Association Mapping of Drought Tolerance Traits
Source: PLoS One. 2015 May 13;10(5):e0122165. doi: 10.1371/journal.pone.0122165 (PMC4430295; doi:10.1371/journal.pone.0122165)
Supplement: S4 Table — (PDF) [file pone.0122165.s005.pdf]

S4 Table Reductions in growth and yield parameters due to drought stress in 2011 and 2012

Trait	Av. control-Av stress/Av. control (2011)	Av. control-Av stress/Av. control (2012)
GY	0.242603	0.292573
PY	0.195992	0.221489
PHI	0.06268	0.100175
TF	0.016072	0.018806
PH	-0.04107	-0.0429
PL	0.032522	-0.01463
PD	-0.00934	0.063409
PN	0.076175	0.044995
TPP	0.032632	0.117044
BY	0.232047	0.197031
GHI	0.0175	0.128675
TGW	0.103549	0.160548
GNPP	0.082316	0.123839
GNPM	0.155964	0.16169

GY, Grain Yield; PY, Panicle yield; PHI, Panicle harvest Index; TF, Flowering time; PH, Plant height; PL, Panicle length; PD, Panicle diameter; PNPH, Panicle number; TPP, Tiller per plant; BY, Biomass yield; GHI, Grain harvest index; TGW, Thousand grain weight; GNPP, Grain number per panicle; GNPM, Grain number per M^2
