# Supplementary material for: Exploring Potential of Pearl Millet Germplasm Association Panel for Association Mapping of Drought Tolerance Traits
Source: PLoS One. 2015 May 13;10(5):e0122165. doi: 10.1371/journal.pone.0122165 (PMC4430295; doi:10.1371/journal.pone.0122165)
Supplement: S5 Table — (PDF) [file pone.0122165.s006.pdf]

S5 Table Correlation among traits under control conditions

Character		GY	PY	PHI	TF	PH	PL	PD	PN	TPP	BY	GHI	TGW	GNPP
PY	2011	0.877**												
	2012	0.961**												
PHI	2011	0.531**	0.069											
	2012	0.717**	0.509**											
TF	2011	-0.098	-0.061	-0.095										
	2012	-0.305**	-0.277**	-0.258**										
PH	2011	0.042	0.067	-0.018	0.577**									
	2012	-0.176**	-0.161*	-0.159*	0.615**									
PL	2011	0.007	0.031	-0.032	0.443**	0.434**								
	2012	0.08	0.149*	-0.103	0.244**	0.362**								
PD	2011	0.329**	0.335**	0.087	-0.098	0.031	-0.245**							
	2012	0.17**	0.202**	0.044	0.067	0.155*	0.063							
PN	2011	-0.193**	-0.137*	-0.19**	-0.484**	-0.392**	-0.519**	-0.183**						
	2012	0.141*	0.168**	-0.002	-0.534**	-0.558**	-0.389**	-0.322**						
TPP	2011	-0.171**	-0.109	-0.193**	-0.515**	-0.447**	-0.52**	-0.089	0.935**					
	2012	0.072	0.093	-0.022	-0.537**	-0.537**	-0.334**	-0.255**	0.863**					
BY	2011	0.597**	0.718**	-0.019	0.368**	0.472**	0.098	0.191**	-0.158*	-0.2**				
	2012	0.454**	0.506**	0.132*	0.271**	0.417**	0.154	0.061	-0.046**	-0.205**				
GHI	2011	0.702**	0.466**	0.651**	-0.446**	-0.367**	-0.07	0.231**	-0.101	-0.036	-0.131*			
	2012	0.783**	0.708**	0.697**	-0.511**	-0.487**	-0.023	0.15*	0.184**	0.22**	-0.183**			
TGW	2011	0.005	-0.133*	0.234**	-0.211**	0.003	-0.139*	0.239**	-0.047	-0.025	-0.122	0.084*		
	2012	-0.018	-0.036	0.004	-0.202**	-0.027	-0.143*	0.217**	-0.044	-0.027	0.003	-0.023		
GNPP	2011	0.536**	0.502**	0.246**	0.438**	0.346**	0.499**	0.167**	-0.744**	-0.717**	0.419**	0.296**	-0.394**	
	2012	0.498**	0.473**	0.408**	0.308**	0.333**	0.41**	0.259**	-0.618**	-0.582**	0.28**	0.357**	-0.402**	
GNPM	2011	0.754**	0.759**	0.229**	0.076	0.036	0.095	0.077	-0.099	-0.108	0.557**	0.452**	-0.634**	0.657**
	2012	0.84**	0.821**	0.587**	-0.141*	-0.137*	0.134*	0.011	0.162**	0.089	0.384**	0.659**	-0.535**	0.623**

** Values significant at p<0..001, *Values significant at p<0.01

GY, Grain Yield; PY, Panicle yield; PHI, Panicle harvest Index; TF, Flowering time; PH, Plant height; PL, Panicle length; PD, Panicle diameter; PN, Panicle number; TPP, Tiller per plant; BY, Biomass yield; GHI, Grain harvest index; TGW, Thousand grain weight; GNPP, Grain number per panicle; GNPM, Grain number per M^2
