# Supplementary material for: Exploring Potential of Pearl Millet Germplasm Association Panel for Association Mapping of Drought Tolerance Traits
Source: PLoS One. 2015 May 13;10(5):e0122165. doi: 10.1371/journal.pone.0122165 (PMC4430295; doi:10.1371/journal.pone.0122165)
Supplement: S6 Table — (PDF) [file pone.0122165.s007.pdf]

S6 Table Correlation among traits under late drought stress treatments

Character		GY	PY	PHI	TF	PH	PL	PD	PN	TPP	BY	GHI	TGW	GNPP	GNPM	LR
PY	2011	0.872**														
	2012	0.971**														
PHI	2011	0.742**	0.337**													
	2012	0.824**	0.678**													
TF	2011	-0.030	-0.012	-0.023												
	2012	0.156*	0.148*	0.135												
PH	2011	-0.002	0.03	-0.016	0.48**											
	2012	0.292**	0.289**	0.242**	0.698**											
PL	2011	0.275**	0.289**	0.136*	0.388**	0.347**										
	2012	0.222**	0.241**	0.14*	0.224**	0.318**										
PD	2011	0.075	0.106	0.007	-0.178**	0.069	-0.111									
	2012	0.225**	0.224**	0.193**	0.162*	0.256**	0.146*									
PN	2011	- 0.213**	-0.131*	-0.244**	-0.509**	-0.377**	-0.473**	-0.126*								
	2012	-0.02	0.018	-0.144*	-0.471**	-0.478**	-0.451**	-0.422**								
TPP	2011	-0.189**	-0.168**	-0.135*	-0.503**	-0.36**	-0.396**	-0.109	0.871**							
	2012	-0.069	-0.038	0.159*	-0.473**	-0.486**	-0.447**	-0.408**	0.917**							
BY	2011	0.575**	0.691**	0.184**	0.288**	0.428**	0.209**	0.042	-0.089	-0.197**						
	2012	0.74	0.762**	0.522**	0.585**	0.628**	0.213**	0.173**	-0.139*	-0.204**						
GHI	2011	0.792**	0.554**	0.778**	-0.25**	-0.313**	0.18**	0.073	0.199**	-0.079	-0.028					
	2012	0.717**	0.65**	0.711**	-0.371**	-0.23**	0.089	0.153*	0.1	0.104	0.082					
TGW	2011	0.222**	0.034	0.381**	-0.201**	0.022	-0.027	0.131**	-0.047	-0.017	0.072	0.231**				
	2012	0.243**	0.237**	0.217**	-0.057	0.14*	-0.057	0.181**	-0.038	-0.022	0.25**	0.125*				
GNPP	2011	0.549**	0.49**	0.402**	0.447**	0.253**	0.519**	0.053	-0.753**	-0.672**	0.314	0.434**	-0.28**			
	2012	0.677**	0.641**	0.627**	0.445**	0.457**	0.481**	0.334**	-0.559**	-0.565**	0.567**	0.422**	-0.198**			
GNPM	2011	0.72**	0.743**	0.384**	0.116	-0.003	0.277**	-0.039	-0.15*	-0.146*	0.475	0.523**	-0.489**	0.695**		
	2012	0.845**	0.824**	0.688**	0.197**	0.197**	0.249**	0.106	0.014	-0.044	0.605**	0.62**	-0.286**	0.778**		
LR	2011	-0.106*	-0.084	-0.068	0.419**	0.389**	0.155*	-0.18**	-0.184**	-0.148*	0.064	-0.173**	-0.265**	0.19**	0.091	
	2012	-0.518**	-0.519**	-0.398**	-0.111	-0.146*	-0.176**	-0.151*	0.121	0.173**	-0.436**	-0.301**	-0.388**	-0.303**	-0.306**	
SG	2011	0.147*	0.092	0.145*	-0.023	-0.125*	0.092	-0.19**	0	-0.016	0.138	0.073	0.234**	-0.018	-0.034	-0.452**
	2012	0.154*	0.131*	0.167**	0.487**	0.369**	0.091	-0.035	0.169**	-0.211**	0.486**	-0.289**	0.306**	0.101	0.008	-0.422**

** Values significant at p<0.001

*Values significant at p<0.01

GY, Grain Yield; PY, Panicle yield; PHI, Panicle harvest Index; TF, Flowering time; PH, Plant height; PL, Panicle length; PD, Panicle diameter; PNPH, Panicle number; TPP, Tiller per plant; BY, Biomass yield; GHI, Grain harvest index; TGW, Thousand grain weight; GNPP, Grain number per panicle; GNPM, Grain number per M^2; LR,Leaf rolling ; SG, Stay green
